# Supplementary material for: 2′–5′ oligoadenylate synthetase‑like 1 (OASL1) protects against atherosclerosis by maintaining endothelial nitric oxide synthase mRNA stability
Source: Nat Commun. 2022 Nov 4;13:6647. doi: 10.1038/s41467-022-34433-z (PMC9636244; doi:10.1038/s41467-022-34433-z)
Supplement: Supplementary file 1 — Supplementary Information [file 41467_2022_34433_MOESM1_ESM.pdf]

Supplementary Information

2′−5′ Oligoadenylate Synthetase-Like 1 (OASL1) Protects Against Atherosclerosis by Maintaining Endothelial Nitric Oxide Synthase mRNA Stability

Tae Kyeong Kim et al.

Supplementary Figures 1–19

Supplementary Table 1

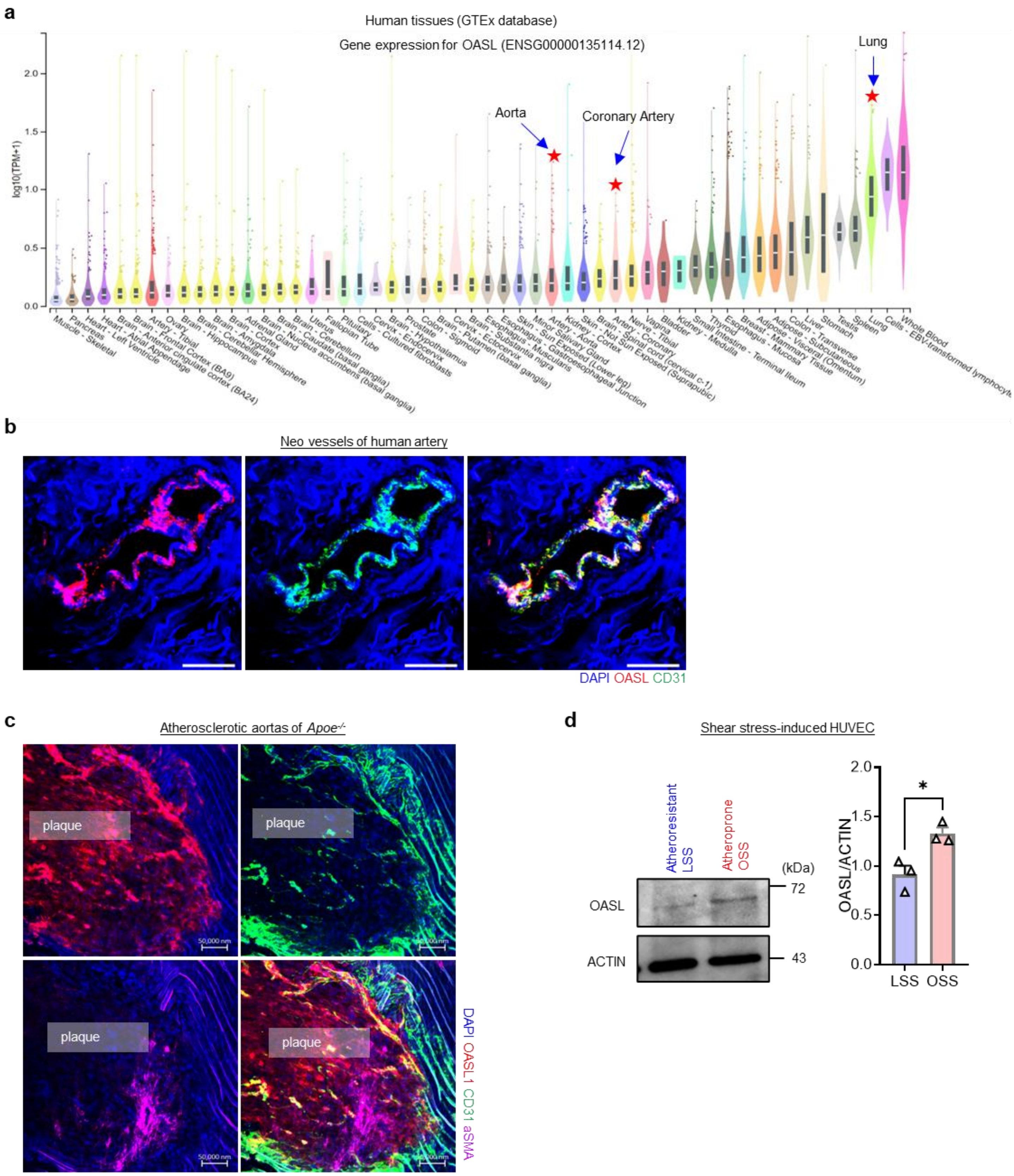
a
Human tissues (GTEx database)
Gene expression for OASL (ENSG00000135114.12)
log10(TPM+1)
Aorta
Coronary Artery
Lung
b
Neo vessels of human artery
DAPI OASL CD31
c
Atherosclerotic aortas of Apoe-/-
plaque
DAPI OASL1 CD31 aSMA
d
Shear stress-induced HUVEC
Atheroresistant LSS
Atheroprone OSS
(kDa)
OASL
ACTIN
72
43
OASL/ACTIN
LSS
OSS
*

Supplementary Figure 1. Human OASL and murine OASL1 are expressed in vascular endothelial cells. a Mining of the GTEx database (https://www.gtexportal.org/home/) for RNA-sequencing data on OASL expression in normal human tissues. EC-enriched tissues, including coronary artery (sample size = 240, Median: 0.7513), lung (sample size = 578, Median: 7.679), and aorta (sample size = 432, Median: 0.5658) are highlighted and indicated. TPM, transcripts per million kilobases. Data were retrieved on Sep 06, 2022 (Data Source: GTEx Analysis Release V8 (dbGaP Accession phs000424.v8.p2)). Box plots are shown as median and 25th and 75th percentiles; points are displayed as outliers if they are above or below 1.5 times the interquartile range. **b** Immunofluorescence (IF) staining of OASL protein and $CD31^+$ ECs in human atheroma tissues with plaques. Scale bars, 50 μm. **c** *En face* IF staining for OASL1 and $CD31^+$ ECs and α-SMA^+ SMCs in plaque-containing *Apoe*$^{-/-}$ aortas. Scale bars, 50 μm. **d** Western blot analysis of OASL expression in shear-induced HUVECs (p = 0.0189, n = 3 per group). LSS: laminar shear stress. OSS: oscillatory shear stress. Data in **b** and **c** are representative of 3 independent experiments. Data are presented as means ± SEMs (*$p \leq 0.05$; **d**, two-sided unpaired Student's *t*-test). Source data are provided as a Source Data file.

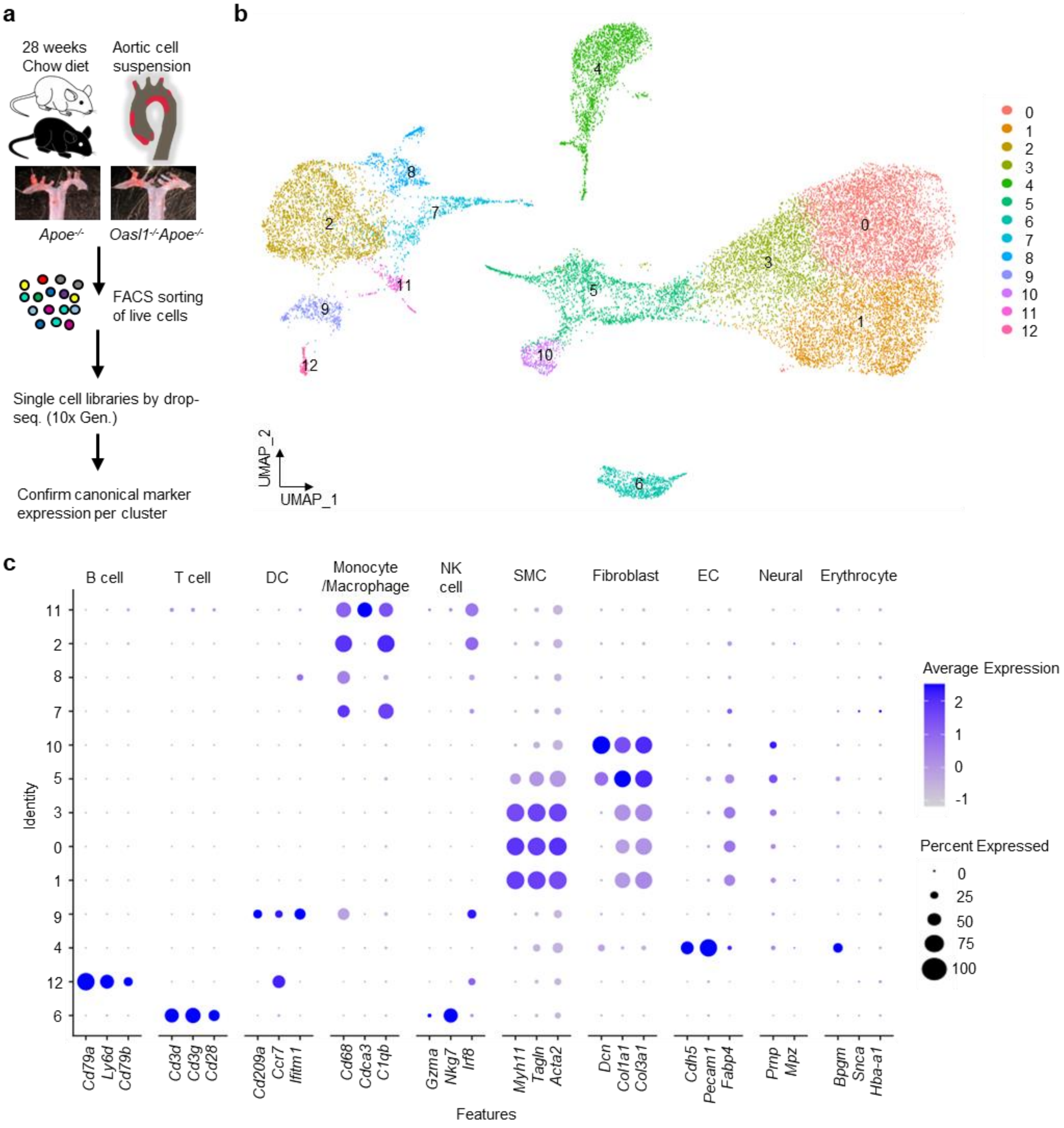

Supplementary Figure 2. scRNA-seq clustering of diverse cell populations from atherosclerotic aortas. a–c *Oasl1*$^{-/-}$*Apoe*$^{-/-}$ and *Apoe*$^{-/-}$ mice were maintained on a normal chow diet (NCD) for 28 weeks to allow the development of atherosclerotic plaques. (**a**) Experimental strategy for scRNA-seq of atherosclerotic aortic single cells. (**b**) Uniform manifold approximation and projection (UMAP) plot

of total aortic cells, colored by the numbering of clusters. (**c**) Dot plot represents the top markers in each aortic cell population. Dot size denotes the proportion of cells within the group expressing each transcript and dot color indicates the average expression level. DC: dendritic cell. NK cell: natural killer cell. SMC: smooth muscle cell. EC: endothelial cell.

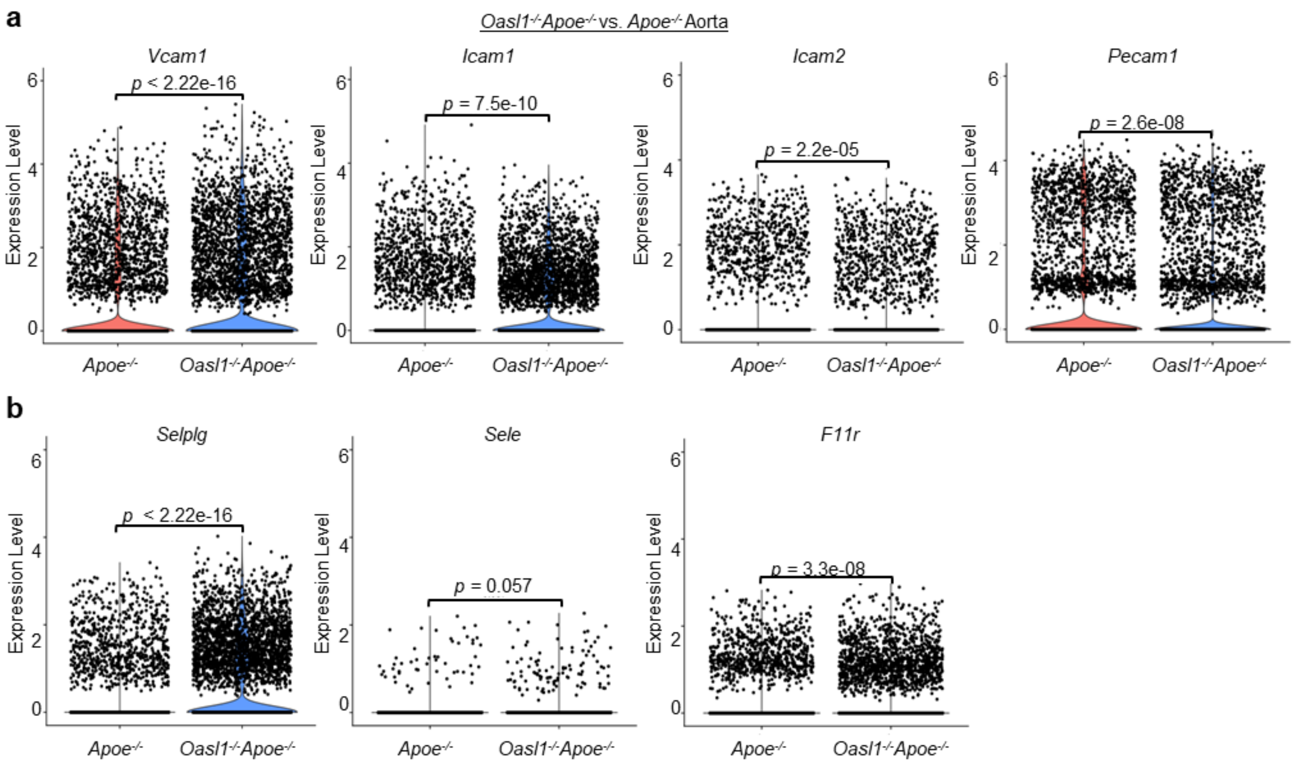

Supplementary Figure 3. *Oasl1* deficiency promotes proinflammatory gene expression in atherosclerotic aortas. a, b Violin plots showing the comparison of adhesion molecule expression in whole aortas. (**a**) *Vcam1*, *Icam1*, *Icam2,* and *Pecam1* expression. (**b**) *Selplg*, *Sele*, and *F11r* expression. Data are presented as means ± SEMs (**a** and **b**, two-sided unpaired Student's *t*-test).

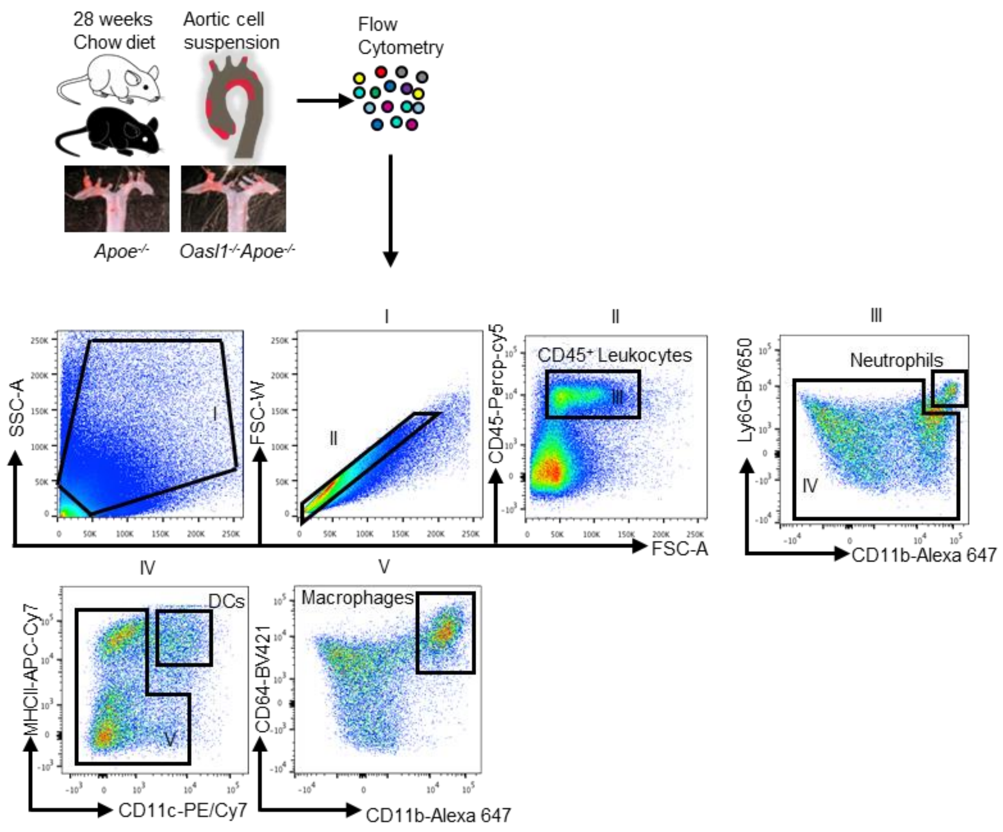

Supplementary Figure 4. Gating strategy for infiltrating immune cells in atherosclerotic aortas. *Oasl1*$^{-/-}$*Apoe*$^{-/-}$ and *Apoe*$^{-/-}$ mice were maintained on a normal chow diet (NCD) for 28 weeks to allow for the development of atherosclerotic plaques. Experimental and gating strategy for flow cytometry analysis of atherosclerotic aortic single cells isolated from the *Oasl1*$^{-/-}$*Apoe*$^{-/-}$ and *Apoe*$^{-/-}$ mice.

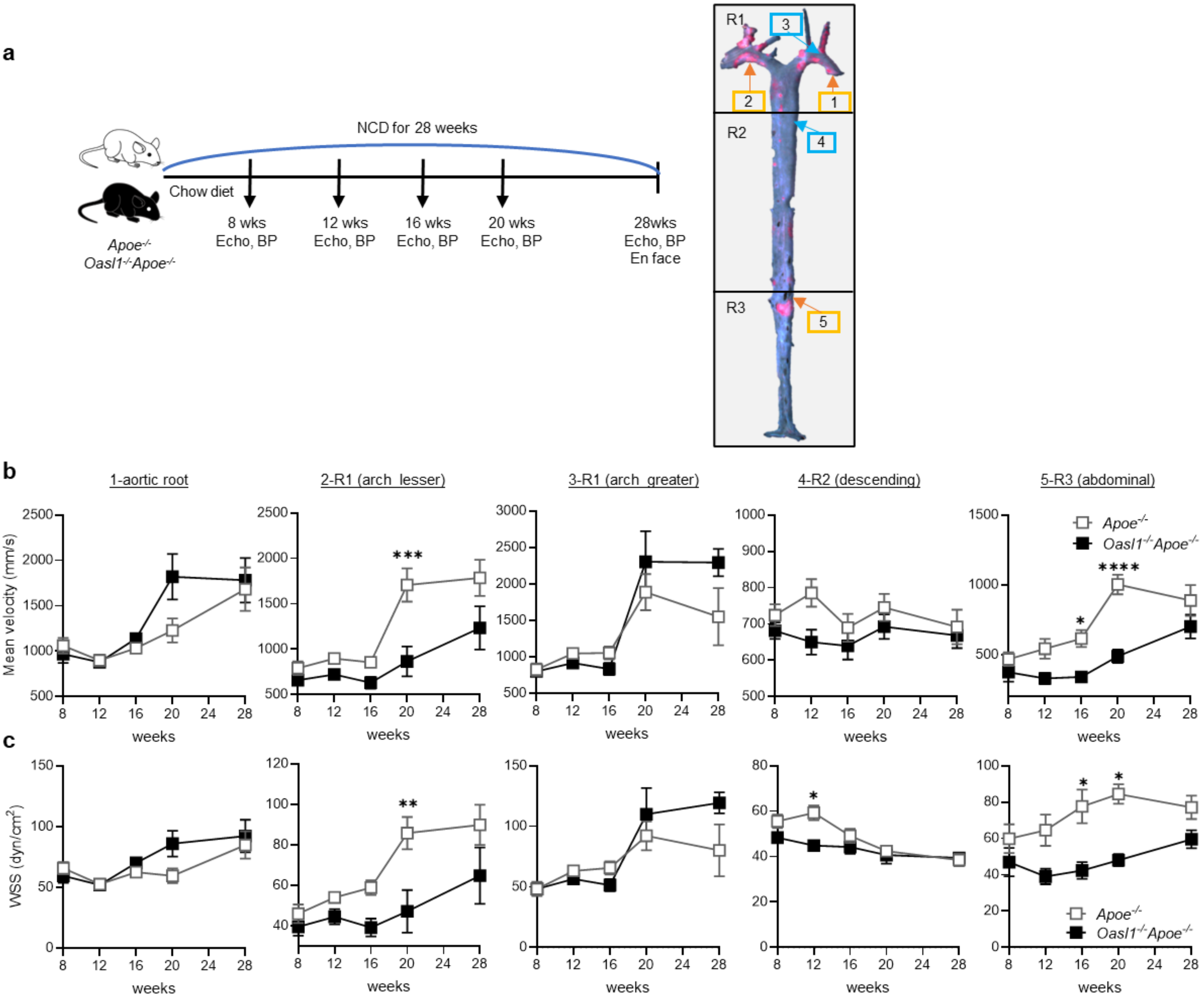

Supplementary Figure 5. ***Oasl1*** **deficiency promotes endothelial dysfunction followed by a reduction of WSS under hyperlipidemic conditions. a–c** Before identifying plaque formation at 28 weeks of NCD, the blood flow dynamics were assessed by serial echocardiography in age-matched *Oasl1*$^{-/-}$*Apoe*$^{-/-}$ and *Apoe*$^{-/-}$ mice (n = 8 for 8–16; n = 6 for 20; n = 5 for 28 weeks per group). (**a**) Left: Experimental scheme. Right: Specific location of the ultrasound measurement in the *en face* of the aorta.

Mean blood velocity (R1 (lesser): $p = 0.0002$, R3: $p = 0.0444$; $p < 0.0001$) (**b**) and endothelial WSS (R1 (lesser): $p = 0.0050$, R3: $p = 0.0118$; $p = 0.0370$) (**c**) at the athero-resistant greater curvature or descending region and athero-prone lesser curvature or abdominal branching point were measured. BP: blood pressure. WSS: wall shear stress. Data are presented as means ± SEMs (*$p \leq 0.05$, **$p \leq 0.01$, ***$p \leq 0.001$, ****$p \leq 0.0001$; **b–c**, two-way ANOVA with Tukey's test for multiple comparisons). Source data are provided as a Source Data file.

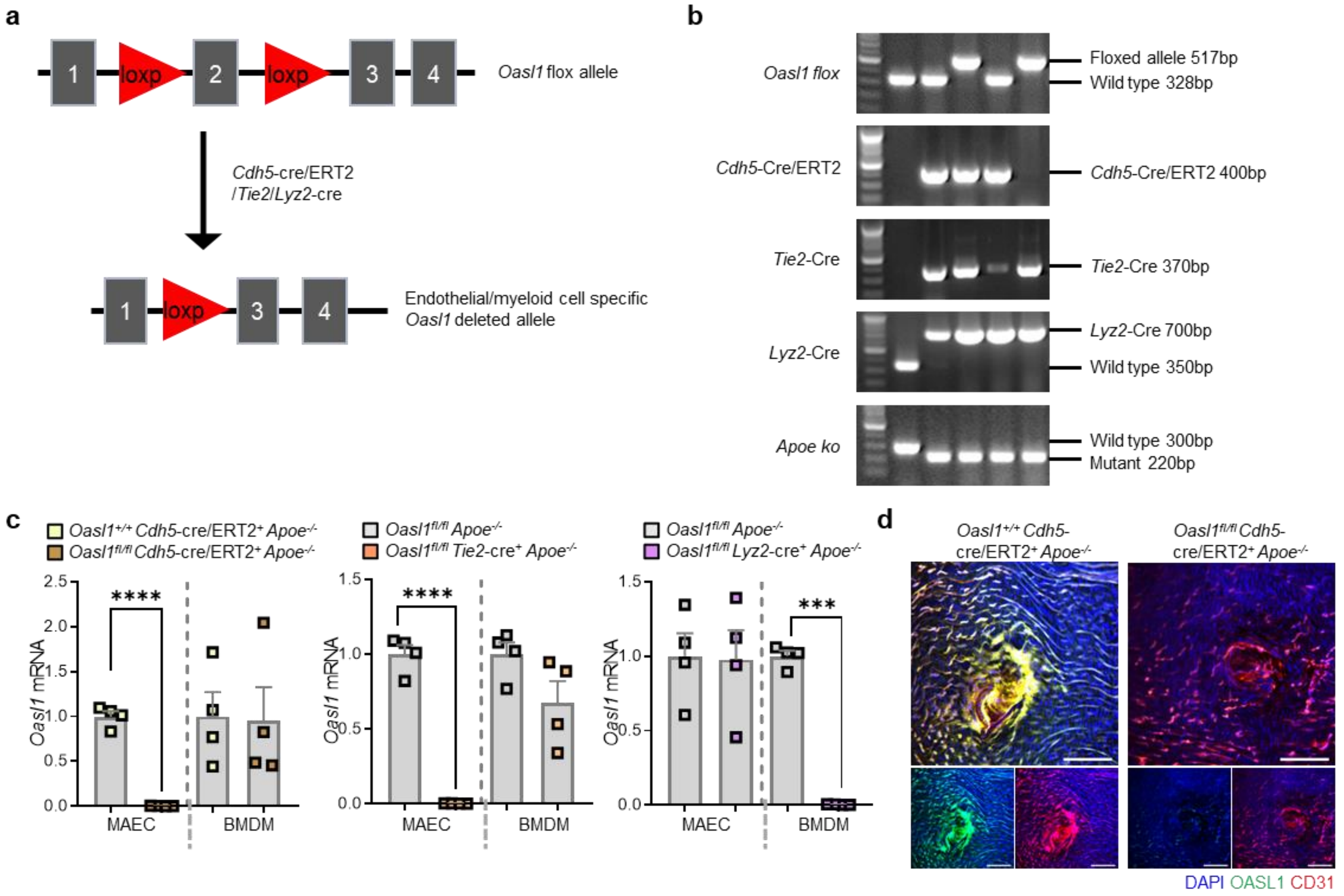

Supplementary Figure 6. Generation and characterization of endothelial cell-specific *Oasl1*-deficient mice. a Scheme for generating cell-specific *Oasl1* knockout mice using the Cre/LoxP system. **b** Genotyping of cell-specific *Oasl1* knockout mice. Molecular weight information is provided. **c** Confirmation of the deletion for *Oasl1* in endothelial cells (ECs) and bone marrow-derived macrophages (BMDMs) isolated from each cre-expressing *Oasl1*$^{fl/fl}$ mouse by qRT-PCR analysis (Cdh5 cre MAEC: $p < 0.0001$, Tie2 cre MAEC: $p < 0.0001$, Lyz2 cre BMDM: $p < 0.0001$, n = 4 per group). *Oasl1* expression in the non-endothelial cell fraction was unaffected, indicating the specific knockout of *Oasl1* in ECs. **d** *En face* IF staining for OASL1 and CD31$^+$ ECs in the endothelium of aortas isolated from *Oasl1*$^{fl/fl}$ Cdh5-cre/ERT2$^+$ *Apoe*$^{-/-}$ and *Oasl1*$^{+/+}$ Cdh5-cre/ERT2$^+$ *Apoe*$^{-/-}$ mice. Scale bar, 50 µm. BMDM: bone marrow-derived macrophages. Data are presented as means ± SEMs (***$p \leq 0.001$, ****$p \leq 0.0001$; **c**, two-sided unpaired Student's *t*-test). Source data are provided as a Source Data file.

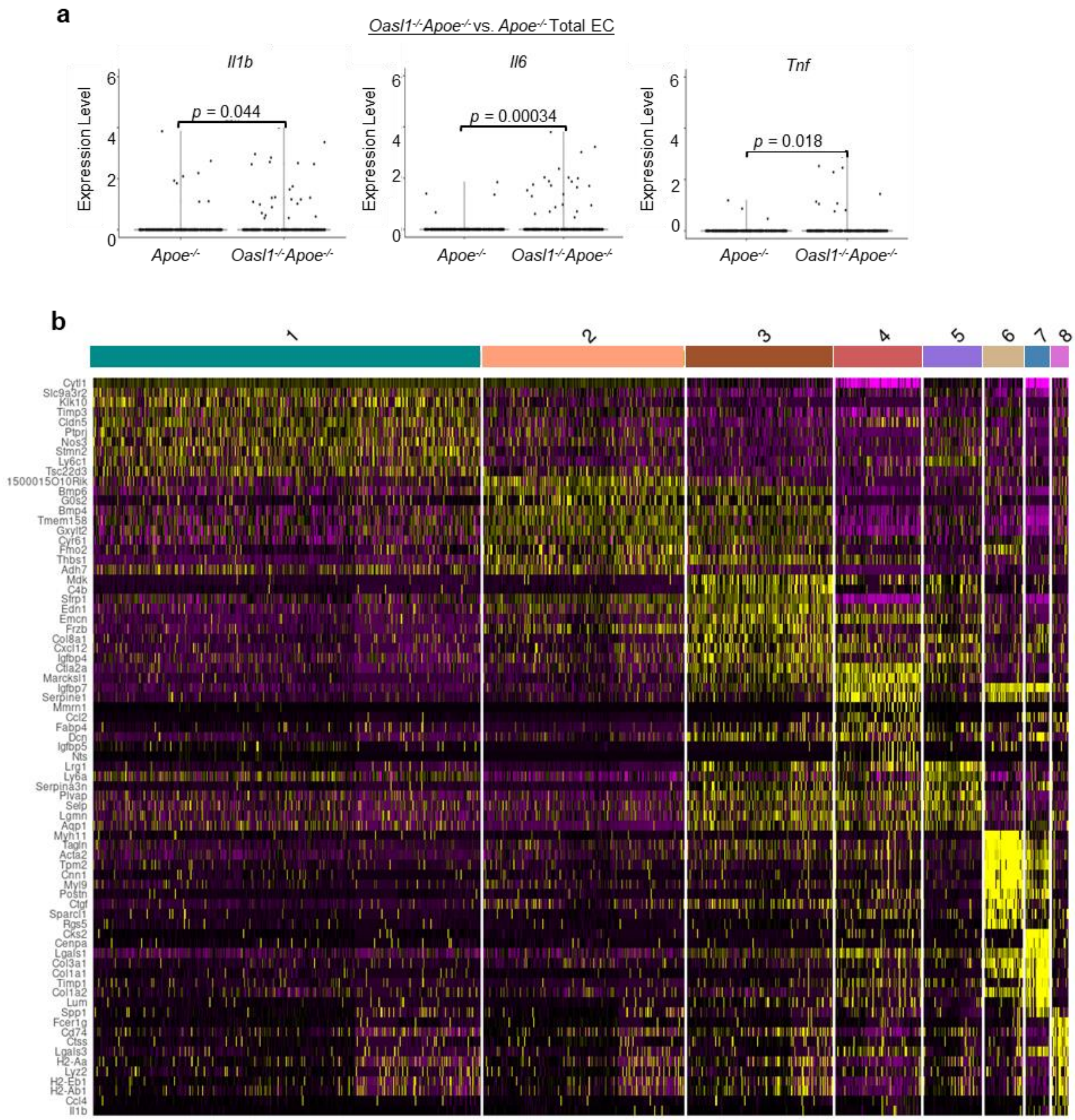

Supplementary Figure 7. ***Oasl1*-deficient ECs exhibit inflammatory characteristics. a** Violin plot comparing the expression of the inflammatory cytokines, *Il1b*, *Il6,* and *Tnf*, in the EC cluster of whole aortas isolated from *Oasl1*$^{-/-}$*Apoe*$^{-/-}$ and *Apoe*$^{-/-}$ mice. **b** Heat map representation showing different gene expression patterns among the EC sub-clusters. Data are presented as means ± SEMs (**a**, two-sided unpaired Student's *t*-test).

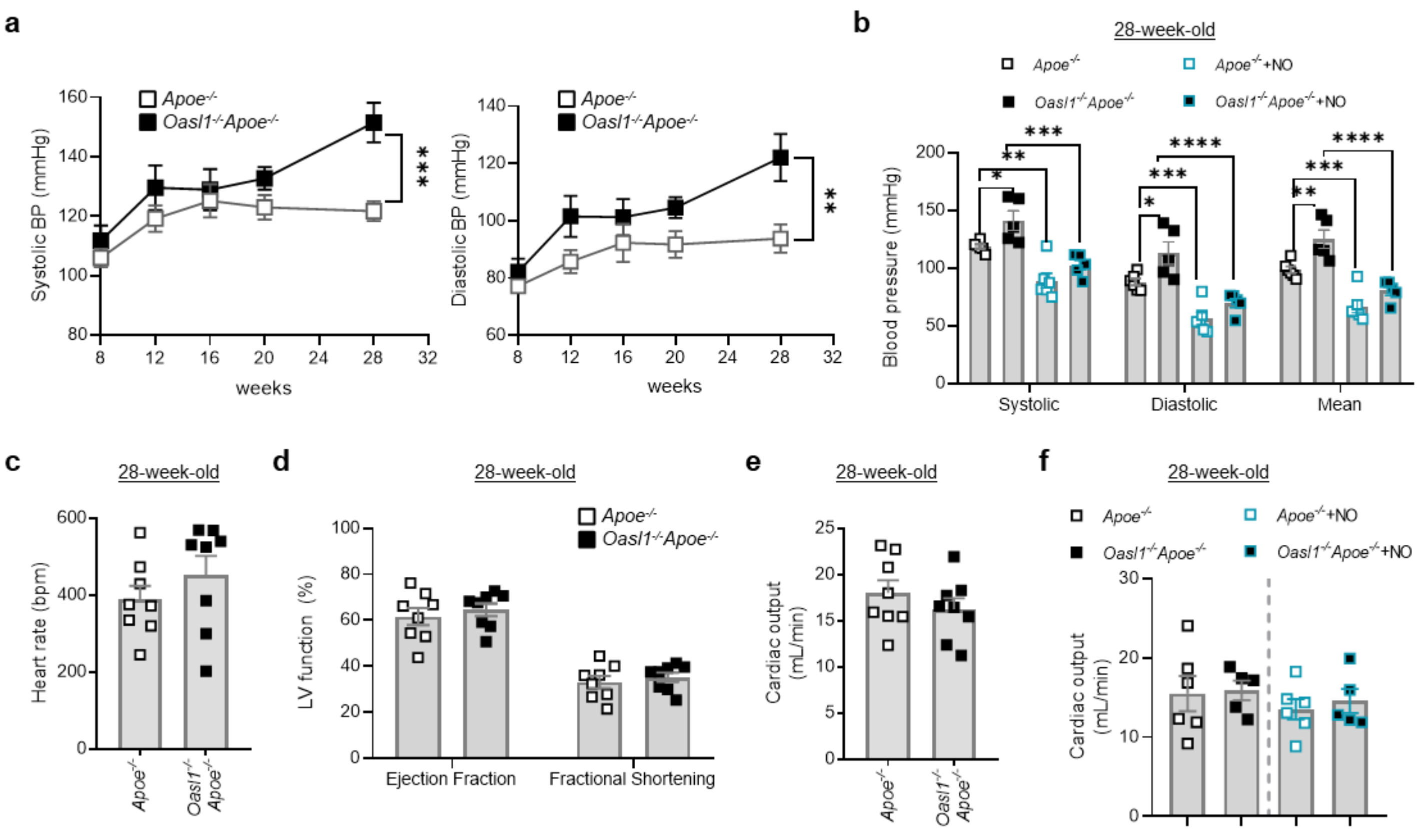

Supplementary Figure 8. ***Oasl1*** **deficiency increases blood pressure dependent on NO levels without affecting cardiac function. a–f** *Apoe*$^{-/-}$ and *Oasl1*$^{-/-}$*Apoe*$^{-/-}$ mice were fed an NCD for 28 weeks to allow for atherosclerotic conditions to develop. (**a**) Left: Systolic blood pressure (BP; p = 0.0009) and Right: diastolic BP (p = 0.0030) change measured using tail-cuff techniques (n = 7 per group). (**b**) BP measurement after nitric oxide (NO) supplementation by DETANONATE injection into 28-week-old mice (Systolic: $p = 0.0309$; $p = 0.0013$; $p = 0.0001$, Diastolic: $p = 0.0123$; $p = 0.0005$; $p < 0.0001$, Mean: $p = 0.0071$; $p = 0.0006$; $p < 0.0001$, n = 6 for *Apoe*$^{-/-}$, *Apoe*$^{-/-}$+NO; n = 5 for *Oasl1*$^{-/-}$*Apoe*$^{-/-}$, *Oasl1*$^{-/-}$*Apoe*$^{-/-}$+NO). **c** Heart rate measured using a tail-cuff system (n = 8 per group). **d, e** Left ventricle (LV) function was measured by serial echocardiography and calculated using Vevo software (n = 8 per group). (**d**) Ejection fraction and fractional shortening. (**e**) Cardiac output. **f** Cardiac output measurement after NO supplementation by DETANONATE injection into 28-week-old mice (n

= 6 for *Apoe*$^{-/-}$, *Apoe*$^{-/-}$+NO; n = 5 for *Oasl1*$^{-/-}$*Apoe*$^{-/-}$, *Oasl1*$^{-/-}$*Apoe*$^{-/-}$+NO). Data are presented as means ± SEMs (*$p \leq 0.05$, **$p \leq 0.01$, ***$p \leq 0.001$, ****$p \leq 0.0001$; **a**, two-way ANOVA with Sidak's test; **b** and **f**, two-way ANOVA with Tukey's test for multiple comparisons; **c**–**e**, two-sided unpaired Student's *t*-test). Source data are provided as a Source Data file.

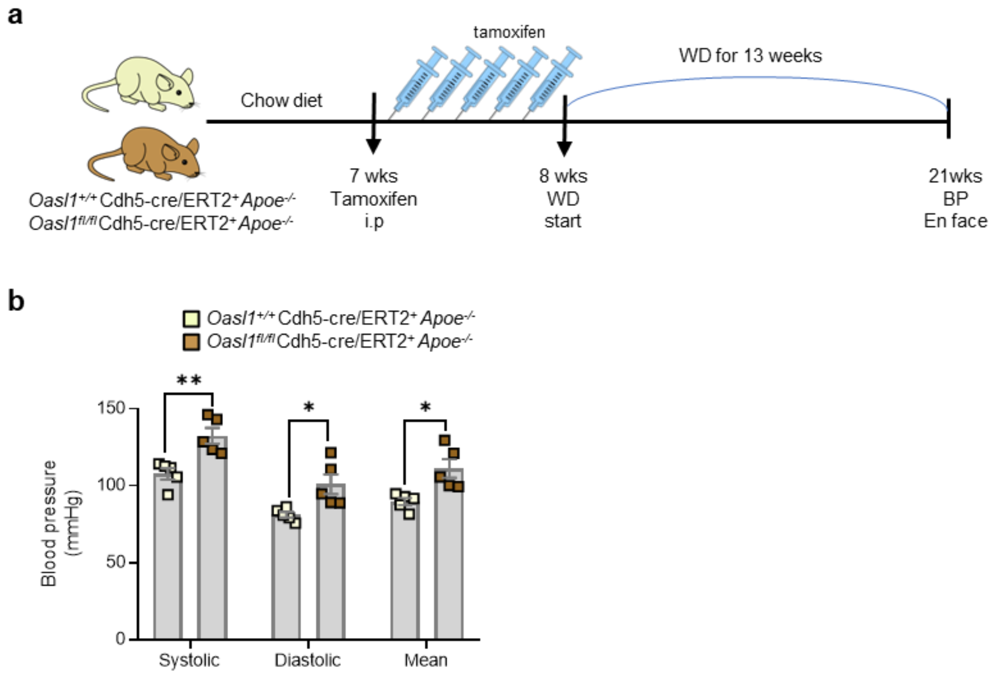

Supplementary Figure 9. Endothelial *Oasl1* deficiency increases blood pressure following plaque formation. a–b Blood pressure (BP) was measured in age-matched *Oasl1*$^{fl/fl}$ *Cdh5-cre/ERT2*$^{+}$ *Apoe*$^{-/-}$ and *Oasl1*$^{+/+}$ *Cdh5-cre/ERT2*$^{+}$ *Apoe*$^{-/-}$ mice after being fed a western diet (WD) for 13 weeks. (**a**) Experimental scheme. (**b**) Systolic, diastolic, and mean BP change measured using tail-cuff techniques (Systolic: $p = 0.0044$, Diastolic: $p = 0.0188$, Mean: $p = 0.0106$, n = 5 per group). Data are presented as means ± SEMs (*$p \leq 0.05$, **$p \leq 0.01$; **b**, two-sided unpaired Student's *t*-test). Source data are provided as a Source Data file.

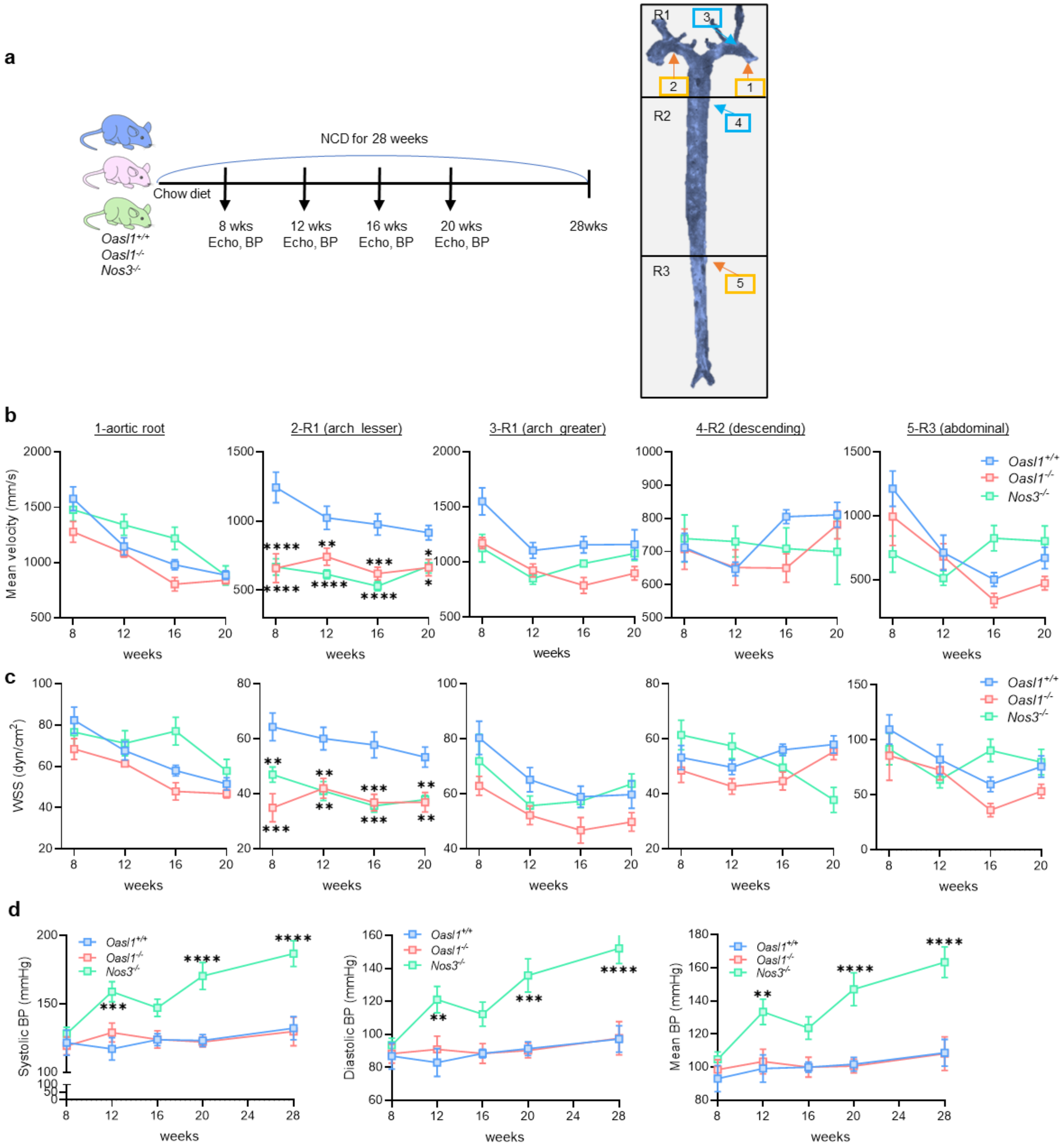
a
NCD for 28 weeks
Chow diet
8 wks
Echo, BP
12 wks
Echo, BP
16 wks
Echo, BP
20 wks
Echo, BP
28wks
Oasl1+/+
Oasl1-/-
Nos3-/-
R1
R2
R3
b
1-aortic root
2-R1 (arch lesser)
3-R1 (arch greater)
4-R2 (descending)
5-R3 (abdominal)
Mean velocity (mm/s)
weeks
c
WSS (dyn/cm2)
d
Systolic BP (mmHg)
Diastolic BP (mmHg)
Mean BP (mmHg)

Supplementary Figure 10. *Oasl1* deficiency reduces blood velocity and WSS. a–d Blood pressure (BP) and blood flow dynamics were measured in age-matched *Oasl1*$^{-/-}$, *Nos3*$^{-/-}$ and *Oasl1*$^{+/+}$ mice. (**a**) Left: Experimental scheme. Right: Specific location for ultrasound measurement in the *en face* of the aorta. Mean blood velocity (R1 (lesser) 8: $p < 0.0001$; $p < 0.0001$, 12: $p = 0.0090$; $p < 0.0001$, 16: $p = 0.0007$; $p < 0.0001$, 20: $p = 0.0218$; $p = 0.0292$) (**b**) and endothelial WSS (R1 (lesser) 8: $p < 0.0001$; $p = 0.0030$, 12: $p = 0.0019$; $p = 0.0010$, 16: $p = 0.0003$; $p = 0.0001$, 20: $p = 0.0052$; $p = 0.0091$) (**c**) at the athero-resistant greater curvature or descending region and athero-prone lesser curvature or abdominal branching point as assessed by serial echocardiography (n = 8 per group except n = 5 for 8-week-old *Oasl1*$^{-/-}$). (**d**) Left: Systolic BP (*Nos3*$^{-/-}$ vs. *Oasl1*$^{+/+}$ 12: $p = 0.0004$, 20: $p < 0.0001$, 28: $p < 0.0001$), Middle: diastolic BP (*Nos3*$^{-/-}$ vs. *Oasl1*$^{+/+}$ 12: $p = 0.0011$, 20: $p = 0.0001$, 28: $p < 0.0001$) and Right: mean BP (*Nos3*$^{-/-}$ vs. *Oasl1*$^{+/+}$ 12: $p = 0.0034$, 20: $p < 0.0001$, 28: $p < 0.0001$) change measured using tail-cuff techniques (n = 7 per group). WSS: wall shear stress. Data are presented as means ± SEMs (*$p \leq 0.05$, **$p \leq 0.01$, ***$p \leq 0.001$, ****$p \leq 0.0001$; **b–d**, two-way ANOVA with Tukey's test for multiple comparisons). Source data are provided as a Source Data file.

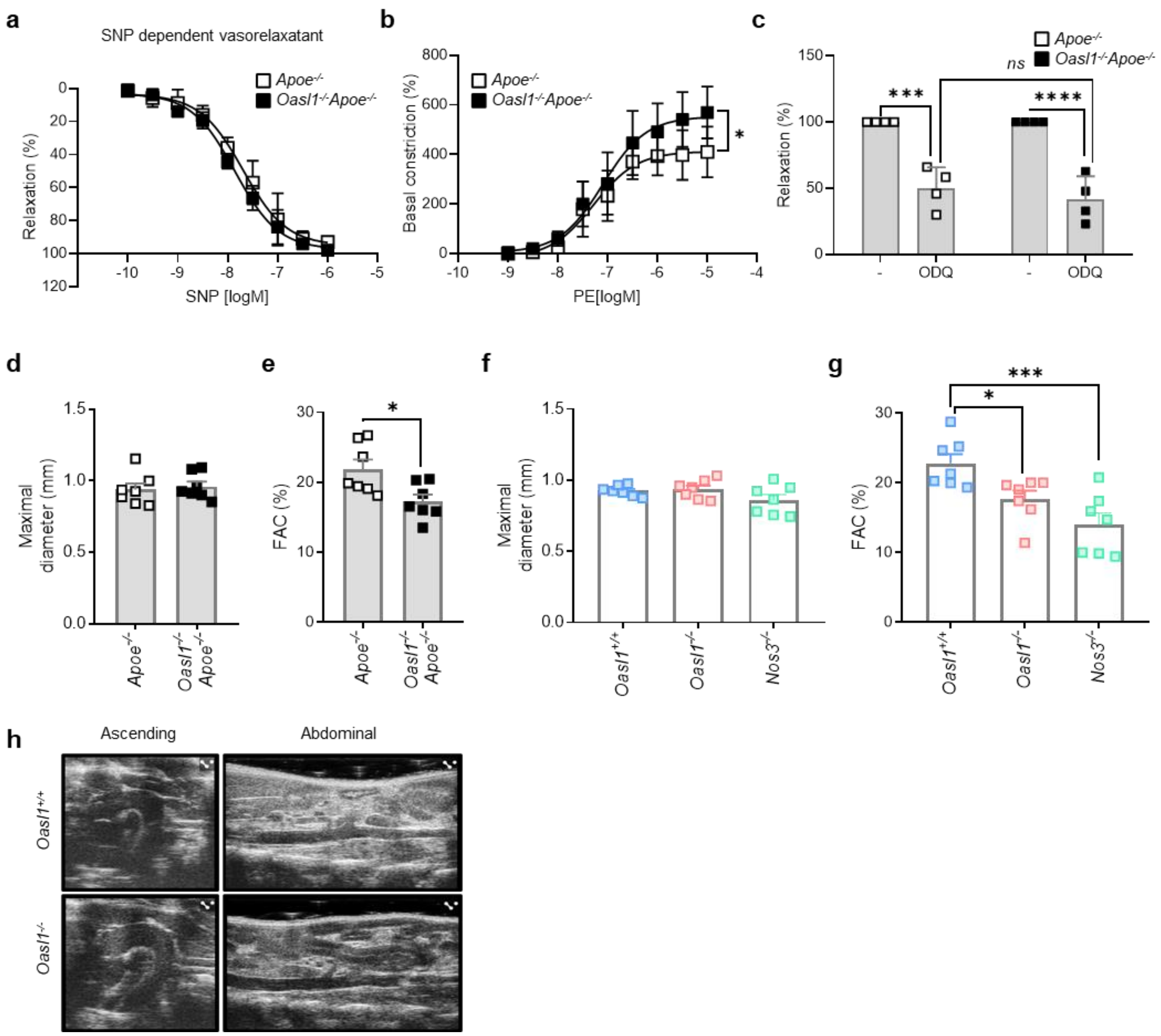

Supplementary Figure 11. *Oasl1* deficiency reduces vascular tension and increases constriction. a–c The cumulative vascular response of the aortic rings isolated from *Oasl1*$^{-/-}$*Apoe*$^{-/-}$ and *Apoe*$^{-/-}$ mice as measured by myography (n = 4 per group). (**a**) Relaxation response to sodium nitroprusside (SNP). (**b**) Constriction response to phenylephrine (PE; $p = 0.0109$). (**c**) The percentage of relaxation response in 1H-[1,2,4]oxadiazolo[4,3-a]quinoxalin-1-one (ODQ) treatment compared with the control (*Apoe*$^{-/-}$: $p = 0.0004$, *Oasl1*$^{-/-}$*Apoe*$^{-/-}$: $p < 0.0001$, n = 4 per group). **d**, **e** *Apoe*$^{-/-}$ and *Oasl1*$^{-/-}$*Apoe*$^{-/-}$

mice were fed an NCD for 16 to 20 weeks and subjected to ultrasound measurements (n = 7 per group). (**d**) Maximal diameter. (**e**) The percentage of fractional area change (FAC; $p = 0.0176$). **f–h** *Oasl1*$^{-/-}$, *Nos3*$^{-/-}$ and *Oasl1*$^{+/+}$ mice were fed an NCD for 16 to 20 weeks and subjected to ultrasound measurements. (**f**) Maximal diameter (n = 7 per group). (**g**) The percentage of FAC ($p = 0.0474$; $p = 0.0009$, n = 7 per group). (**h**) Vascular geometry. Data are presented as means ± SEMs except **a–c**; means ± SDs ($*p \leq 0.05$, $***p \leq 0.001$, $****p \leq 0.0001$; Nonlinear fit data in **a** and **b** were calculated using a Sigmoidal dose-response and two-sided unpaired Student's *t*-test; **c**, two-way ANOVA with Sidak's test; **d** and **e**, two-sided unpaired Student's *t*-test; **f** and **g**, one-way ANOVA with Tukey's test for multiple comparisons). Source data are provided as a Source Data file.

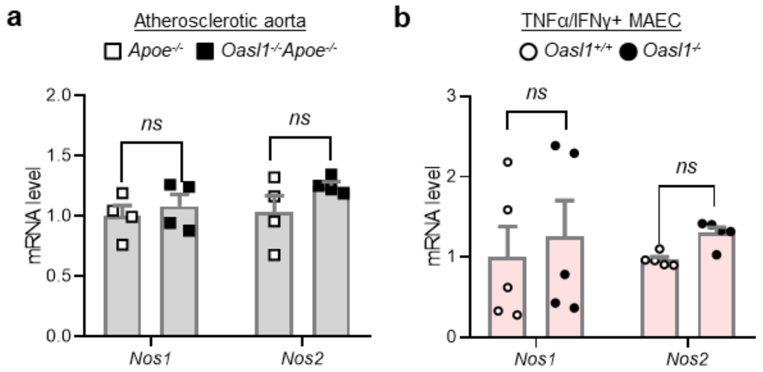

Supplementary Figure 12. *Oasl1* deficiency has little effect on the expression of other *Nos* genes. *Nos1 and Nos2* expression was measured in atherosclerotic aortas (n = 4 per group) (**a**) or in MAECs stimulated with TNFα and IFNγ (n = 5 per group) (**b**) by quantitative PCR analysis. Data are presented as means ± SEMs (**a** and **b**, two-sided unpaired Student's t-test). Source data are provided as a Source Data file.

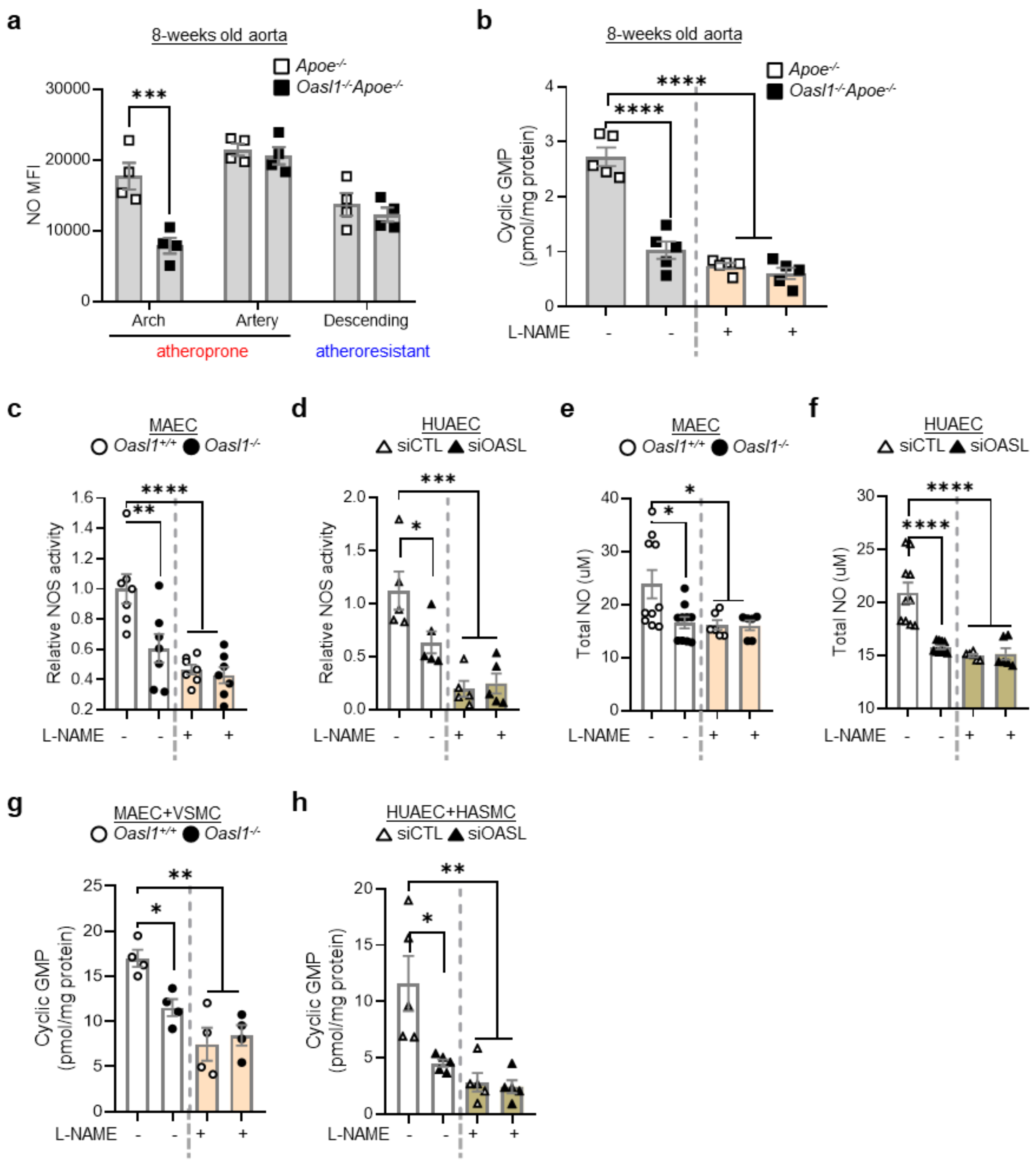

Supplementary Figure 13. *Oasl1* deficiency reduces eNOS/NO bioavailability followed by a decrease in aortic cGMP levels. a–b Aorta tissues were isolated from *Apoe*$^{-/-}$ and *Oasl1*$^{-/-}$*Apoe*$^{-/-}$ mice fed an NCD for 8 weeks. (**a**) Quantitation of nitric oxide (NO) MFI results according to regional specificity ($p = 0.0041$, n = 4 per group). (**b**) Cyclic guanosine monophosphate (cGMP) level in aortas following incubation with or without N^G-nitro-L-arginine methyl ester (L-NAME) for 24 h (vs.

Apoe$^{-/-}$ −L-NAME: $p < 0.0001$; $p < 0.0001$, n = 5 per group). Analysis of NOS enzymatic activity in MAECs isolated from *Oasl1*$^{+/+}$ and *Oasl1*$^{-/-}$ mice (vs. *Oasl1*$^{+/+}$ −L-NAME: $p = 0.0018$; $p < 0.0001$; $p < 0.0001$, n = 7 per group) (**c**) and in HUAECs transfected with siCTL or siOASL (vs. siCTL −L-NAME: $p = 0.0379$; $p = 0.0003$; $p = 0.0006$, n = 5 per group) (**d**) following treatment with L-NAME. Total nitric oxide (NO) levels in the supernatants of ECs including MAECs isolated from *Oasl1*$^{+/+}$ and *Oasl1*$^{-/-}$ mice (vs. *Oasl1*$^{+/+}$ −L-NAME: $p = 0.0239$; $p = 0.0416$; $p = 0.0396$, n = 10 for non-treat; n = 6 for L-NAME) (**e**) and HUAECs transfected with siCTL or siOASL (vs. siCTL −L-NAME: $p < 0.0001$; $p < 0.0001$; $p < 0.0001$, n = 10 for non-treat; n = 6 for L-NAME) (**f**) following treatment with L-NAME for 24 h. Cyclic GMP (cGMP) levels in mouse VSMCs co-cultured with MAECs (vs. *Oasl1*$^{+/+}$ −L-NAME: $p = 0.0324$; $p = 0.0010$; $p = 0.0022$, n = 4 per group) (**g**) and human VSMCs co-cultured with HUAECs (vs. siCTL −L-NAME: $p = 0.0110$; $p = 0.0023$; $p = 0.0016$, n = 5 per group) (**h**) following incubation with or without L-NAME for 24 h. Data are presented as means ± SEMs (*$p \leq 0.05$, **$p \leq 0.01$, ***$p \leq 0.001$, ****$p \leq 0.0001$; **a**, two-sided unpaired Student's t-test; **b**–**d**, **g** and **h**, two-way ANOVA with Tukey's test; **e** and **f**, one-way ANOVA with Tukey's test for multiple comparisons). Source data are provided as a Source Data file.

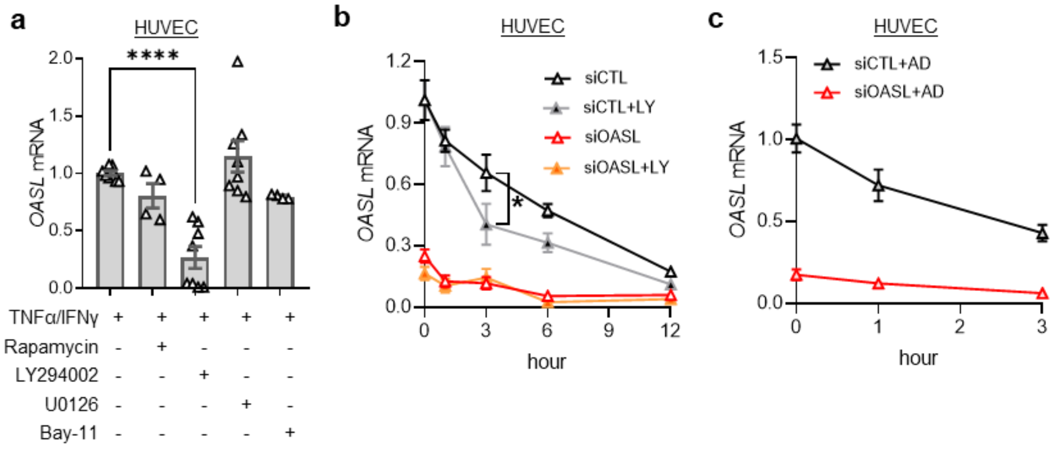

Supplementary Figure 14. *OASL* expression is suppressed by PI3K/Akt inhibition. qRT-PCR analysis of *OASL* mRNA in (**a**) HUVECs pretreated with the mTOR inhibitor, rapamycin, PI3K inhibitor, LY294002, MEK1/2 inhibitor, U0126, or NF-κB inhibitor, Bay-11 (20 μmol/L each) 1 h before stimulation with TNF-α and IFN-γ for 3 h ($p < 0.0001$, n = 8 for DMSO, LY294002, U0126; n = 4 for Rapamycin, Bay11), and (**b**) siCTL- or siOASL-transfected HUVECs, pretreated with the PI3K inhibitor, LY294002 (LY), and monitored over time (siCTL vs. siCTL+LY 3h: $p = 0.0096$, n = 4 per group). **c** Confirmation of *OASL* mRNA levels in siCTL- or siOASL-transfected HUVECs. *OASL* mRNA was measured over time in the presence of actinomycin D (AD), which was added to block transcription (n = 4 per group). Data are presented as means ± SEMs (*$p \leq 0.05$, ****$p \leq 0.0001$; **a**, one-way ANOVA with Bonferroni's test; **b** and **c**, two-way ANOVA with Tukey's test for multiple comparisons). Source data are provided as a Source Data file.

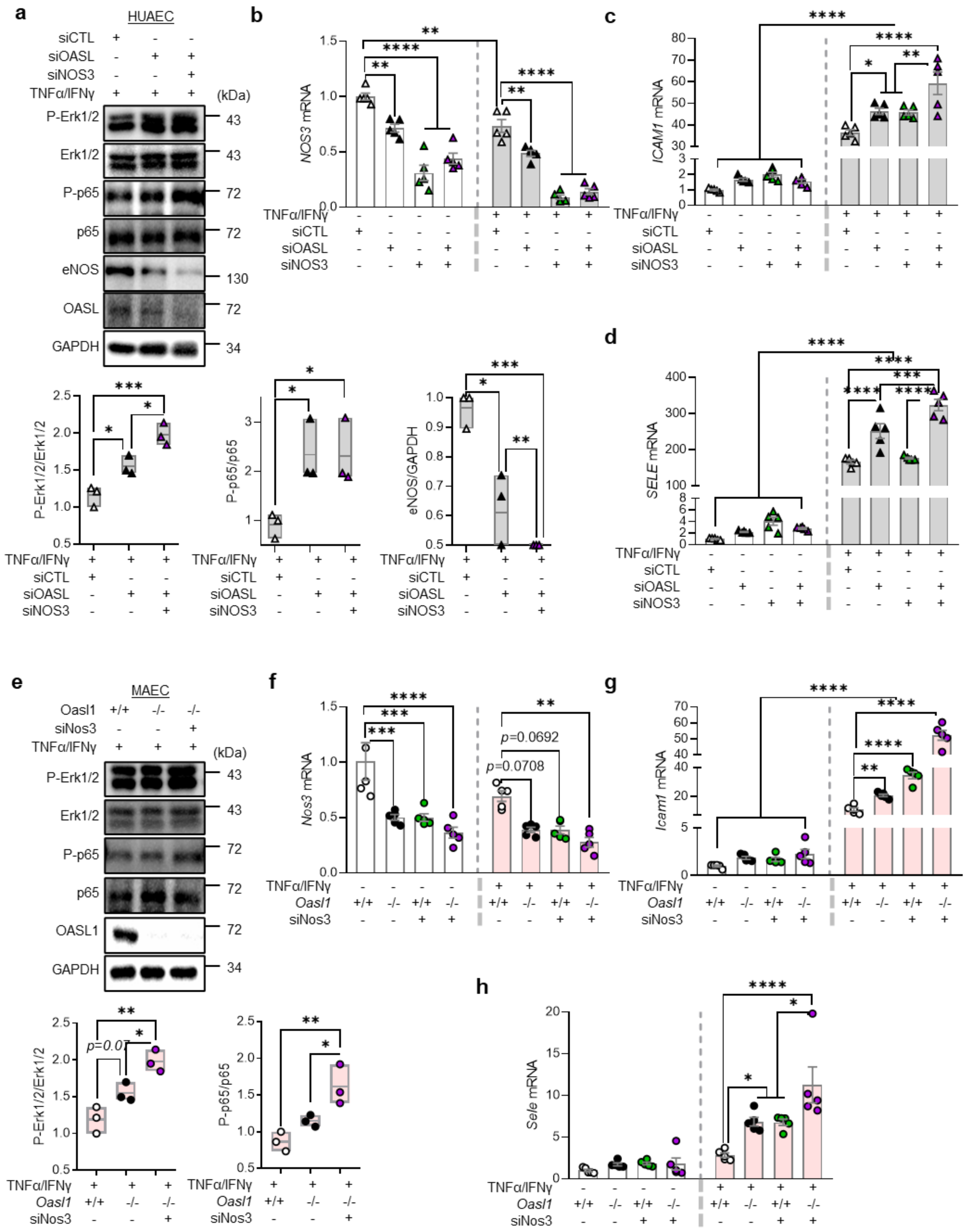

a
HUAEC
siCTL
siOASL
siNOS3
TNFα/IFNγ
(kDa)
P-Erk1/2
Erk1/2
P-p65
p65
eNOS
OASL
GAPDH
43
72
130
34
P-Erk1/2/Erk1/2
P-p65/p65
eNOS/GAPDH
b
NOS3 mRNA
c
ICAM1 mRNA
d
SELE mRNA
e
MAEC
Oasl1
siNos3
OASL1
f
Nos3 mRNA
p=0.0692
p=0.0708
g
Icam1 mRNA
h
Sele mRNA
p=0.07

Supplementary Figure 15. ***Nos3*** **knockdown potentiates endothelial inflammation in** ***Oasl1*****-deficient ECs. a–d** siCTL- or siOASL-transfected HUAECs were co-transfected with siNOS3 and stimulated with TNF-α and IFN-γ. (**a**) Top: Western blot analysis of Erk1/2, p65, and eNOS expression. Bottom: Quantitation of band density (P-Erk1/2: $p = 0.0315$; $p = 0.0009$; $p = 0.0217$, P-p65: $p = 0.0455$; $p = 0.0485$, eNOS: $p = 0.0181$; $p = 0.0003$; $p = 0.0065$). Quantitative PCR analysis of *NOS3* (non siOASL: $p = 0.0011$; siNOS3, siOASL/siNOS3: $p < 0.0001$, non vs. T/I siCTL: $p = 0.0026$, T/I siOASL: $p = 0.0063$; siNOS3, siOASL/siNOS3: $p < 0.0001$) (**b**) and the adhesion molecules *ICAM1* (non vs. T/I: $p < 0.0001$, T/I siOASL: $p = 0.0298$; siOASL/siNOS3: $p < 0.0001$; siOASL or siNOS3 vs. siOASL/siNOS3: $p = 0.0020$; $p = 0.0011$) (**c**) and *SELE* (non vs. T/I: $p < 0.0001$, T/I siOASL, siOASL/siNOS3: $p < 0.0001$; siOASL or siNOS3 vs. siOASL/siNOS3: $p = 0.0001$; $p < 0.0001$) (**d**) (n = 5 per group). **e–h** MAECs isolated from *Oasl1*$^{+/+}$ and *Oasl1*$^{-/-}$ mice were co-transfected with siNos3 and stimulated with TNF-α and IFN-γ. (**e**) Top: Western blot analysis of Erk1/2 and p65 expression. Bottom: Quantitation of band density (P-Erk1/2: $p = 0.0019$; $p = 0.0336$, P-p65: $p = 0.0054$; $p = 0.0438$). Quantitative PCR analysis of *Nos3* (non *Oasl1*$^{-/-}$, siNos3: $p = 0.0003$; *Oasl1*$^{-/-}$/siNos3: $p < 0.0001$, T/I *Oasl1*$^{-/-}$/siNos3: $p = 0.0042$) (**f**) and the adhesion molecules *Icam1* (non vs. T/I: $p < 0.0001$, T/I *Oasl1*$^{-/-}$: $p = 0.0010$; siNos3, *Oasl1*$^{-/-}$/siNos3: $p < 0.0001$) (**g**) and *Sele* (T/I *Oasl1*$^{-/-}$: $p = 0.0389$; siNos3: $p = 0.0455$, *Oasl1*$^{-/-}$/siNos3: $p < 0.0001$; *Oasl1*$^{-/-}$ vs. *Oasl1*$^{-/-}$/siNos3: $p = 0.0174$; siNos3 vs. *Oasl1*$^{-/-}$/siNos3: $p = 0.0147$) (**h**) (n = 5 per group). Data in **a** and **e** are representative of 3 independent experiments. **a** and **e**, Box plots are shown as median of each value and the interquartile range (IQR, the range between the 25th and 75th percentiles). Data are presented as means ± SEMs (*$p \leq 0.05$, **$p \leq 0.01$, ***$p \leq 0.001$, ****$p \leq 0.0001$; **a** and **e**, one-way ANOVA with Tukey's test; **b–d**, **f–h**, two-way ANOVA with Tukey's test for multiple comparisons). Source data are provided as a Source Data file.

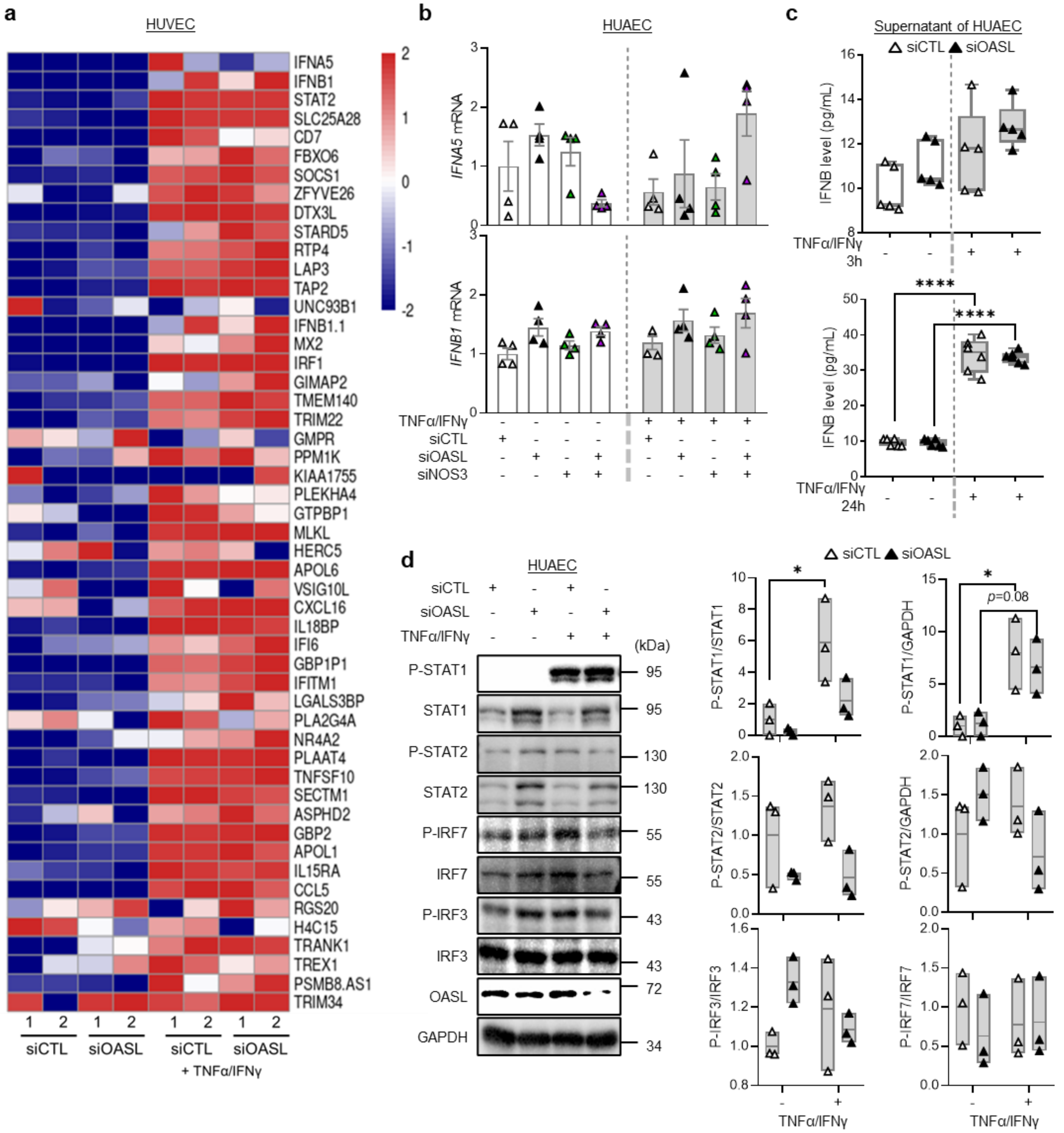

a
HUVEC
IFNA5
IFNB1
STAT2
SLC25A28
CD7
FBXO6
SOCS1
ZFYVE26
DTX3L
STARD5
RTP4
LAP3
TAP2
UNC93B1
IFNB1.1
MX2
IRF1
GIMAP2
TMEM140
TRIM22
GMPR
PPM1K
KIAA1755
PLEKHA4
GTPBP1
MLKL
HERC5
APOL6
VSIG10L
CXCL16
IL18BP
IFI6
GBP1P1
IFITM1
LGALS3BP
PLA2G4A
NR4A2
PLAAT4
TNFSF10
SECTM1
ASPHD2
GBP2
APOL1
IL15RA
CCL5
RGS20
H4C15
TRANK1
TREX1
PSMB8.AS1
TRIM34
siCTL
siOASL
+ TNFα/IFNγ
b
HUAEC
IFNA5 mRNA
IFNB1 mRNA
TNFα/IFNγ
siCTL
siOASL
siNOS3
c
Supernatant of HUAEC
siCTL siOASL
IFNB level (pg/mL)
TNFα/IFNγ 3h
TNFα/IFNγ 24h
****
d
HUAEC
siCTL
siOASL
TNFα/IFNγ
(kDa)
P-STAT1
STAT1
P-STAT2
STAT2
P-IRF7
IRF7
P-IRF3
IRF3
OASL
GAPDH
P-STAT1/STAT1
P-STAT1/GAPDH
p=0.08
P-STAT2/STAT2
P-STAT2/GAPDH
P-IRF3/IRF3
P-IRF7/IRF7

Supplementary Figure 16. Endothelial *OASL* knockdown shows no differences in type I IFN signaling. **a** Heatmap of type I interferon (IFN)-stimulated genes from bulk RNA-seq analysis performed on siCTL- or siOASL-transfected HUVECs stimulated with or without TNFα and IFNγ (n = 2 per group). **b** Quantitative PCR analysis of *IFNA5* and *IFNB1* in siCTL- or siOASL-transfected HUAECs co-transfected with siNOS3 and stimulated with TNF-α and IFN-γ (n = 4 per group). **c–d** siCTL- or siOASL-transfected HUVECs were stimulated with TNF-α and IFN-γ. (c) Secreted IFNB levels in the supernatants of HUAECs. Top: stimulated for 3 h (n = 5 per group). Bottom: stimulated for 24 h (siCTL: $p < 0.0001$, siOASL: $p < 0.0001$, n = 6 per group). (**d**) Left: Western blot analysis of STAT1, STAT2, IRF7, and IRF3 expression. Right: Quantification of band density (P-STAT1/STAT1: $p = 0.0305$, P-STAT1/GAPDH: $p = 0.0121$; $p = 0.0420$). **c** and **d**, Box plots are shown as median of each value and the IQR; whiskers indicate 1.5 times the IQR. Data in **d** are representative of 3 independent experiments. Data are presented as means ± SEMs (*$p \leq 0.05$, ****$p \leq 0.0001$; **b** and **c**, two-way ANOVA with Tukey's test; **d**, two-way ANOVA with Sidak's test for multiple comparisons). Source data are provided as a Source Data file.

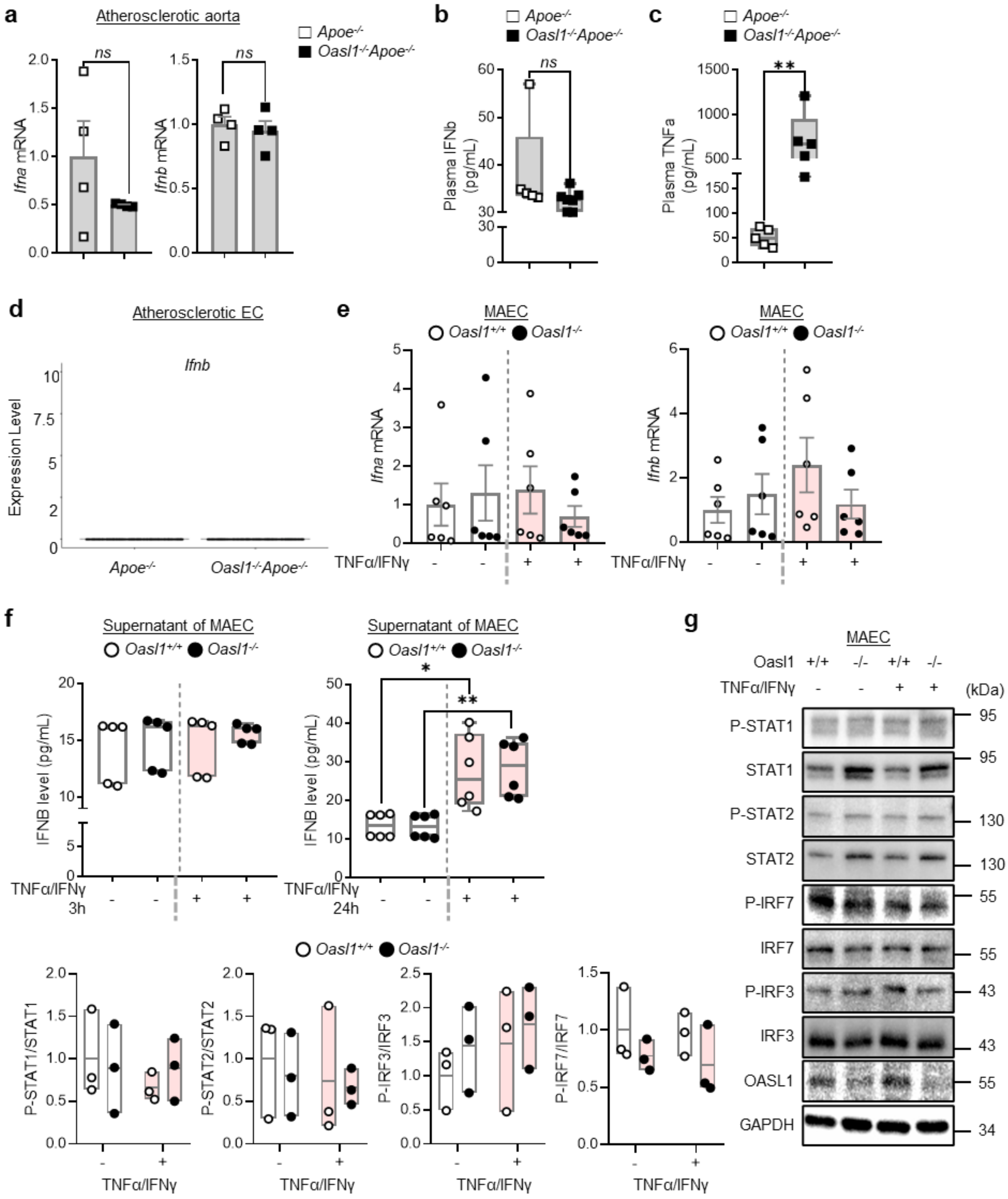

Supplementary Figure 17. Endothelial *Oasl1* deficiency shows no differences in type I IFN signaling. a–d *Apoe*$^{-/-}$ and *Oasl1*$^{-/-}$*Apoe*$^{-/-}$ mice were fed an NCD for 28 weeks to allow atherosclerotic conditions to develop. (**a**) Quantitative PCR analysis of type I IFNs, *Ifna* and *Ifnb*, in

atherosclerotic aortas (n = 4 per group). (**b**) Plasma IFNβ levels. (n = 5 for *Apoe*$^{-/-}$; n = 7 for *Oasl1*$^{-/-}$*Apoe*$^{-/-}$) (**c**) Plasma TNFα levels. ($p = 0.0066$, n = 5 per group). (**d**) Violin plot comparing *Ifnb* in the aortic EC cluster of *Oasl1*$^{-/-}$*Apoe*$^{-/-}$ versus *Apoe*$^{-/-}$ mice. **e–g** MAECs isolated from *Oasl1*$^{+/+}$ and *Oasl1*$^{-/-}$ mice and stimulated with TNFα and IFNγ. (**e**) Quantitative PCR analysis of *Ifna* and *Ifnb* (n = 6 per group). (**f**) Secreted IFNβ levels in the supernatants of MAECs. Left: stimulated for 3 h (n = 5 per group). Right: stimulated for 24 h (*Oasl1*$^{+/+}$: $p = 0.0131$, *Oasl1*$^{-/-}$: $p = 0.0072$, n = 6 per group). (**g**) Right: Western blot analysis of STAT1, STAT2, IRF7, and IRF3 expression. Left: Quantitation of band density. **b**, **c**, **e** and **g**, Box plots are shown as median of each value and the IQR; whiskers indicate 1.5 times the IQR. Data in **g** are representative of 3 independent experiments. Data are presented as means ± SEMs (*$p \leq 0.05$, **$p \leq 0.01$; **a–c**, two-sided unpaired Student's *t*-test; **e** and **f**, two-way ANOVA with Tukey's test; **g**, two-way ANOVA with Sidak's test for multiple comparisons). Source data are provided as a Source Data file.

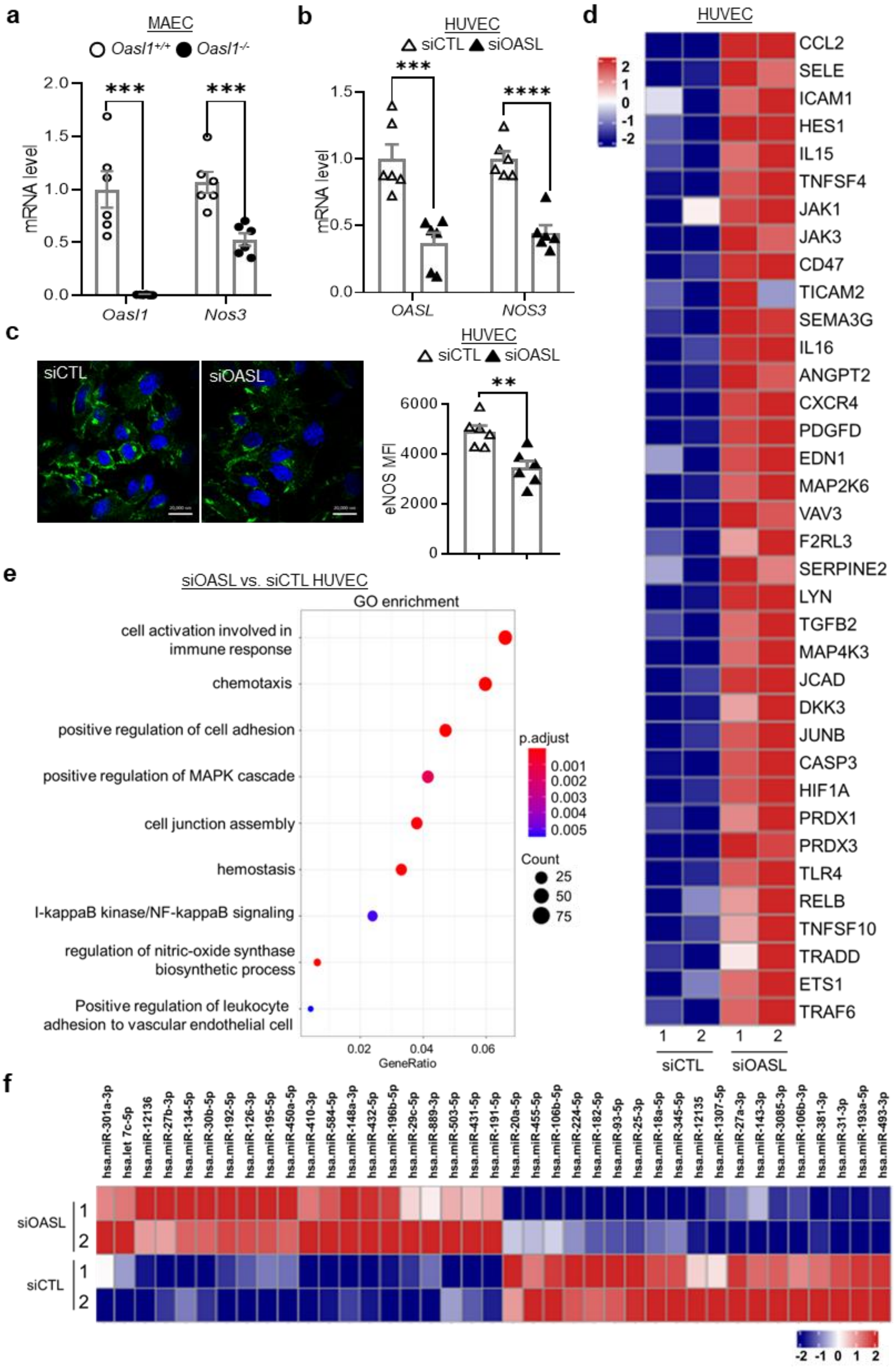
a
MAEC
Oasl1+/+ Oasl1-/-
***
***
mRNA level
2.0
1.5
1.0
0.5
0.0
Oasl1
Nos3
b
HUVEC
siCTL siOASL
***
****
mRNA level
1.5
1.0
0.5
0.0
OASL
NOS3
c
siCTL
siOASL
HUVEC
siCTL siOASL
**
eNOS MFI
6000
4000
2000
0
d
HUVEC
2
1
0
-1
-2
CCL2
SELE
ICAM1
HES1
IL15
TNFSF4
JAK1
JAK3
CD47
TICAM2
SEMA3G
IL16
ANGPT2
CXCR4
PDGFD
EDN1
MAP2K6
VAV3
F2RL3
SERPINE2
LYN
TGFB2
MAP4K3
JCAD
DKK3
JUNB
CASP3
HIF1A
PRDX1
PRDX3
TLR4
RELB
TNFSF10
TRADD
ETS1
TRAF6
1 2 1 2
siCTL siOASL
e
siOASL vs. siCTL HUVEC
GO enrichment
cell activation involved in immune response
chemotaxis
positive regulation of cell adhesion
positive regulation of MAPK cascade
cell junction assembly
hemostasis
I-kappaB kinase/NF-kappaB signaling
regulation of nitric-oxide synthase biosynthetic process
Positive regulation of leukocyte adhesion to vascular endothelial cell
0.02 0.04 0.06
GeneRatio
p.adjust
0.001
0.002
0.003
0.004
0.005
Count
25
50
75
f
hsa.miR-301a-3p
hsa.let 7c-5p
hsa.miR-12136
hsa.miR-27b-3p
hsa.miR-134-5p
hsa.miR-30b-5p
hsa.miR-192-5p
hsa.miR-126-3p
hsa.miR-195-5p
hsa.miR-450a-5p
hsa.miR-410-3p
hsa.miR-584-5p
hsa.miR-148a-3p
hsa.miR-432-5p
hsa.miR-196b-5p
hsa.miR-29c-5p
hsa.miR-889-3p
hsa.miR-503-5p
hsa.miR-431-5p
hsa.miR-191-5p
hsa.miR-20a-5p
hsa.miR-455-5p
hsa.miR-106b-5p
hsa.miR-224-5p
hsa.miR-182-5p
hsa.miR-93-5p
hsa.miR-25-3p
hsa.miR-18a-5p
hsa.miR-345-5p
hsa.miR-12135
hsa.miR-1307-5p
hsa.miR-27a-3p
hsa.miR-143-3p
hsa.miR-3085-3p
hsa.miR-106b-3p
hsa.miR-381-3p
hsa.miR-31-3p
hsa.miR-193a-5p
hsa.miR-493-3p
siOASL 1
2
siCTL 1
2
-2 -1 0 1 2

Supplementary Figure 18. Endothelial *OASL* knockdown reduces *NOS3* levels and activates inflammation. a Quantitative PCR analysis of *Oasl1* and *Nos3* mRNA levels in MAECs isolated from *Oasl1*$^{+/+}$ and *Oasl1*$^{-/-}$ mice (*Oasl1*: $p = 0.0002$, *Nos3*: $p = 0.0009$, n = 6 per group). **b–c** HUVECs were transfected with non-targeting siRNA (siCTL) or OASL-targeting siRNA (siOASL). (**b**) *OASL* and *NOS3* mRNA levels as measured by quantitative PCR (OASL: $p = 0.0008$, NOS3: $p < 0.0001$, n = 6 per group). (**c**) Left: Immunocytochemical detection of eNOS. Scale bar, 20 μm. Right: Quantitation of corresponding MFI values ($p = 0.0031$, n = 6 per group). Scale bar, 20 μm. **d, e** Bulk RNA-seq analysis performed on siCTL- or siOASL-transfected HUVECs (n = 2 for each group). (**d**) Heatmap showing different gene expression patterns between two groups. (**e**) Gene ontology (GO) enrichment analysis of dysregulated biological processes in *OASL*-KD HUVECs. **f** MicroRNA (miRNA)-seq analysis performed on HUVECs transfected with siCTL or siOASL (n = 2 per group), depicted as a heatmap showing differences in miRNA expression patterns between the two groups. Data in **c** are representative of 6 independent experiments. Data are presented as means ± SEMs (**$p \leq 0.01$, ***$p \leq 0.001$, ****$p \leq 0.0001$; **a**–**c**, two-sided unpaired Student's *t*-test; **e**, one-sided Fisher's exact test). Source data are provided as a Source Data file.

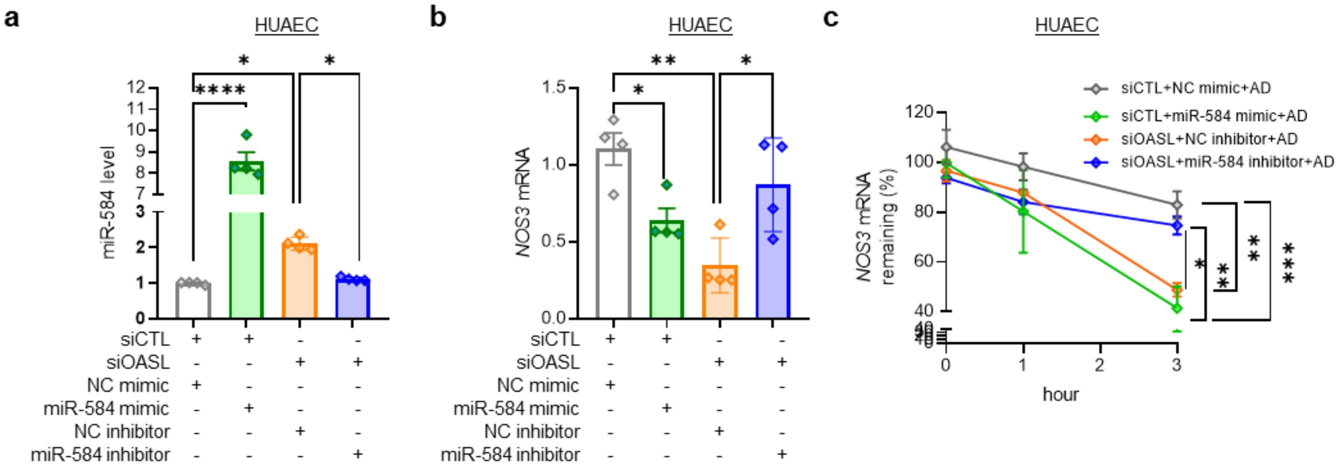

Supplementary Figure 19. Aortic endothelial OASL contributes to *NOS3* mRNA stability by regulating miR-584 levels. qRT-PCR analysis of miR-584 levels ($p < 0.0001$; $p = 0.0159$; $p = 0.0310$, n = 4 per group) (**a**) *NOS3* mRNA ($p = 0.0472$; $p = 0.0018$; $p = 0.0240$, n = 4 per group) (**b**) and *NOS3* mRNA stability in the presence of actinomycin D (AD; 3h NC vs. miR-584 mimic: $p = 0.0004$; NC mimic vs. inhibitor: $p = 0.0046$; miR-584 mimic vs. inhibitor: $p = 0.0059$; NC vs. miR-584 inhibitor: $p = 0.0473$, n = 6 per group) (**c**) in siCTL- or siOASL-transfected HUAECs, post-transfected with 80 nM negative control (NC) mimic, miR-584 mimic, NC inhibitor, or miR-584 inhibitor. NC: negative control. Data are presented as means ± SEMs (*$p \leq 0.05$, **$p \leq 0.01$, ***$p \leq 0.001$, ****$p \leq 0.0001$; **a**, **b**, one-way ANOVA with Tukey's test; **c**, two-way ANOVA with Tukey's test for multiple comparisons). Source data are provided as a Source Data file.

Characteristics	$Apoe^{-/-}$		$Oasl1^{-/-}Apoe^{-/-}$		*p*-value
	Mean	S.E.M.	Mean	S.E.M.	
Plasma glucose (mg/dl)	142.2	10.35	138.8	16.00	0.863
Triglyceride (mg/dl)	88.8	6.07	79.0	10.16	0.432
Total cholesterol (mg/dl)	620.8	21.05	591.2	22.82	0.368
HDL-C (mg/dl)	28.0	3.36	21.4	4.02	0.243
LDL-C (mg/dl)	120.6	8.57	129.2	14.16	0.617
ALB (g/dl)	4.0	0.05	3.8	0.14	0.188
GOT (IU/L)	248.0	13.56	254.0	31.40	0.865
GPT (IU/L)	148.0	20.83	116.0	9.80	0.202

Supplementary Table 1. Plasma lipid levels in $Oasl1^{-/-}Apoe^{-/-}$ and $Apoe^{-/-}$ mice. Plasma lipid profiles in $Oasl1^{-/-}Apoe^{-/-}$ and $Apoe^{-/-}$ mice fed a NCD for 28 weeks (n = 9 per group). Data are presented as means ± SEMs and analyzed using a two-sided unpaired Student's *t*-test.
