# Supplementary material for: 2′–5′ oligoadenylate synthetase‑like 1 (OASL1) protects against atherosclerosis by maintaining endothelial nitric oxide synthase mRNA stability
Source: Nat Commun. 2022 Nov 4;13:6647. doi: 10.1038/s41467-022-34433-z (PMC9636244; doi:10.1038/s41467-022-34433-z)
Supplement: Supplementary file 3 — Description of Additional Supplementary Files [file 41467_2022_34433_MOESM3_ESM.pdf]

**Description of Additional Supplementary Files**

**File name: Supplementary Data 1**

Description: List of antibodies used in this study.

**File name: Supplementary Data 2**

Description: List of qPCR primers used in this study.

**File name: Supplementary Data 3**

Description: List of PCR primer pairs for genotyping used in this study.
