# Supplementary material for: 2′–5′ oligoadenylate synthetase‑like 1 (OASL1) protects against atherosclerosis by maintaining endothelial nitric oxide synthase mRNA stability
Source: Nat Commun. 2022 Nov 4;13:6647. doi: 10.1038/s41467-022-34433-z (PMC9636244; doi:10.1038/s41467-022-34433-z)
Supplement: Supplementary file 7 — Reporting Summary [file 41467_2022_34433_MOESM7_ESM.pdf]

nature portfolio

Corresponding author(s): Goo Taeg Oh

Last updated by author(s): Sep 6, 2022

Reporting Summary

Nature Portfolio wishes to improve the reproducibility of the work that we publish. This form provides structure for consistency and transparency in reporting. For further information on Nature Portfolio policies, see our Editorial Policies and the Editorial Policy Checklist.

Statistics

For all statistical analyses, confirm that the following items are present in the figure legend, table legend, main text, or Methods section.

n/a	Confirmed	
☐	☒	The exact sample size (*n*) for each experimental group/condition, given as a discrete number and unit of measurement
☐	☒	A statement on whether measurements were taken from distinct samples or whether the same sample was measured repeatedly
☐	☒	The statistical test(s) used AND whether they are one- or two-sided<br>*Only common tests should be described solely by name; describe more complex techniques in the Methods section.*
☒	☐	A description of all covariates tested
☐	☒	A description of any assumptions or corrections, such as tests of normality and adjustment for multiple comparisons
☐	☒	A full description of the statistical parameters including central tendency (e.g. means) or other basic estimates (e.g. regression coefficient) AND variation (e.g. standard deviation) or associated estimates of uncertainty (e.g. confidence intervals)
☐	☒	For null hypothesis testing, the test statistic (e.g. *F*, *t*, *r*) with confidence intervals, effect sizes, degrees of freedom and *P* value noted<br>*Give P values as exact values whenever suitable.*
☒	☐	For Bayesian analysis, information on the choice of priors and Markov chain Monte Carlo settings
☒	☐	For hierarchical and complex designs, identification of the appropriate level for tests and full reporting of outcomes
☒	☐	Estimates of effect sizes (e.g. Cohen's *d*, Pearson's *r*), indicating how they were calculated

Our web collection on statistics for biologists contains articles on many of the points above.

Software and code

Policy information about availability of computer code

Data collection	AXIOZOOM V16 (Zeiss) and confocal microscope (LSM780, Zeiss) were used to acquire microscopic image data. ChemiDoc XRS+ (BioRad) or manual development was used to acquire western blot image data. LSR Fortessa (BD Biosciences) was used to acquire flow cytometry data. VisualSonics Vevo 2100 machine (Visual Sonics) was used to acquire echocardiography data.
Data analysis	ZEN2011 (v9.0.2, Zeiss) was used to analyze microscopic image data. ImageLab Software (v6.1, BioRad) and ImageJ (v1.53c, NIH) were used to quantify band intensity. FlowJo (v 10.0.7, FlowJo, LLC) was used to analyze flow cytometry data. Cell Ranger toolkit (v3.1.0, 10x Genomics), R package Seurat (v4.0.3), DoubletFinder (v2.0.3), ggplot2 (v3.3.6), clusterProfiler (v4.4.2), and MAST (v1.20.0) were used to analyze single-cell RNA sequencing data. StringTie-2.1.3b or miRDeep2 (v0.1.3), and R package DESeq2 (v1.34.0) were used to analyze the bulk RNA sequencing data. Vevo LAB software (v5.5.1) including Vevo Strain and Auto LV packages was used to analyze echocardiography data. GraphPad Prism (version 8, GraphPad) was used for statistical analysis.

For manuscripts utilizing custom algorithms or software that are central to the research but not yet described in published literature, software must be made available to editors and reviewers. We strongly encourage code deposition in a community repository (e.g. GitHub). See the Nature Portfolio guidelines for submitting code & software for further information.

Data

Policy information about availability of data

All manuscripts must include a data availability statement. This statement should provide the following information, where applicable:

- Accession codes, unique identifiers, or web links for publicly available datasets
- A description of any restrictions on data availability
- For clinical datasets or third party data, please ensure that the statement adheres to our policy

All data generated during this study are available in the main text or the supplementary information files. Source data are provided with this paper. The single-cell RNA-seq data generated in this study have been deposited in the NCBI Gene Expression Omnibus (GEO) database under accession code GSE186355 [https://www.ncbi.nlm.nih.gov/geo/query/acc.cgi?acc=GSE186355]. The bulk RNA-seq data generated in this study have been deposited in the GEO database under accession code GSE186352 [https://www.ncbi.nlm.nih.gov/geo/query/acc.cgi?acc=GSE186352]. The miRNA-seq data generated in this study have been deposited in the GEO database under accession code GSE186354 [https://www.ncbi.nlm.nih.gov/geo/query/acc.cgi?acc=GSE186354]. GRCh38 [https://ftp.ebi.ac.uk/pub/databases/gencode/Gencode_human/release_38/GRCh38.primary_assembly.genome.fa.gz] and GRCm38 [https://ftp.ebi.ac.uk/pub/databases/gencode/Gencode_mouse/release_M25/GRCm38.primary_assembly.genome.fa.gz] were used for the reference genome.

Field-specific reporting

Please select the one below that is the best fit for your research. If you are not sure, read the appropriate sections before making your selection.

☒ Life sciences ☐ Behavioural & social sciences ☐ Ecological, evolutionary & environmental sciences

For a reference copy of the document with all sections, see nature.com/documents/nr-reporting-summary-flat.pdf

Life sciences study design

All studies must disclose on these points even when the disclosure is negative.

Sample size	The sample size was determined based on previous experiments of our group regarding atherosclerosis and endothelial cells (https://www.ahajournals.org/doi/10.1161/CIRCULATIONAHA.120.046907, https://www.ahajournals.org/doi/10.1161/circresaha.111.245530). We used the sample size of the in vivo and in vitro experiments as n≥3 individual samples for the proper statistical analysis to represent the sufficient reproducibility in vivo and in vitro experiments. One exception is the experiment regarding sequencing using Human umbilical vein EC, which was n=2, but the results were supported by in vitro validation. We did not calculate sample-size calculations to predetermine the sample size. The number of mice used in single-cell RNA sequencing was determined as for the visualization more than 7,000 cells in total. Based on this, the number of pooled mice in each experimental group is as follows: Apoe-/- (n=5) and Oasl1-/- Apoe-/- group (n=5).
Data exclusions	Fig. 8: During bone marrow transplantation and normal chow diet feeding, irradiated and BM-injected Oasl1-/- Apoe-/- recipient mice were failed to live until phenotyping including mean velocity, WSS, FACS and aorta en face.<br>For the quality control to our scRNA-seq analysis, we applied these criteria: (1) mitochondrial genes < 10%. (2) gene counts range from 200 to 5,000.
Replication	Every experiment was repeated at least three times as indicated in the figure legends with exceptions of experiment shown in RNA-seq and miRNA-seq (n=2). All replication of experiments was successful, and the validation was supported by various sequencing.
Randomization	In all experiments used in this study, the samples were divided randomly into each different group.
Blinding	For in vitro experiments, blinding was not applied as all the samples were processed and analyzed with the same protocol and phenotype are distinct for different genotype. For in vivo experiments of plaque phenotyping, echocardiography and blood pressure measurement of mice, investigator were blinded to group allocation during data collection and analysis by randomly numbering the mice.

Reporting for specific materials, systems and methods

We require information from authors about some types of materials, experimental systems and methods used in many studies. Here, indicate whether each material, system or method listed is relevant to your study. If you are not sure if a list item applies to your research, read the appropriate section before selecting a response.

Materials & experimental systems

n/a	Involved in the study
☐	☒ Antibodies
☐	☒ Eukaryotic cell lines
☒	☐ Palaeontology and archaeology
☐	☒ Animals and other organisms
☐	☒ Human research participants
☒	☐ Clinical data
☒	☐ Dual use research of concern

Methods

n/a	Involved in the study
☒	☐ ChIP-seq
☐	☒ Flow cytometry
☒	☐ MRI-based neuroimaging

Antibodies

Antibodies used

Detailed information is described in the Methods section and Supplementary Information.
CD45:PerCP (clone: 30-F11; BioLegend, #103130; Lot: B236192)
CD11b:APC (clone: M1/70; BioLegend, #101212; Lot: B226978)
CD64(FcYRI):BV421 (clone: X54-5/7.1; BioLegend, #139309; Lot: B293980)
Ly6G:BV650 (clone: 1A8; BioLegend, #127641; Lot: B334254)
CD11c:PE/Cy7 (clone: N418; BioLegend, #117318; Lot: B269973)
MHCII(I-A/I-E):APC/Cy7 (clone: M5/114.15.2; BioLegend, #107628; Lot: B257359)
α-SMA (Abcam, ab5694; Lot: GR149079-1)
Alexa Fluor® 488 anti-mouse CD31 (clone: MEC13.3; BioLegend, 102514; Lot: B282351)
Alexa Fluor® 594 anti-mouse CD31 (clone: MEC13.3; BioLegend, 102520; Lot: B280756)
CD31 (Abcam, ab28364; Lot: GR3247742-20)
phospho-Akt (clone: D9E; Cell Signaling Technology, #4060; Lot: 27)
Akt (Cell Signaling Technology, #9272; Lot: 28)
phospho-p65 (Cell Signaling Technology, #3037; Lot: 7)
p65 (clone: D14E12; Cell Signaling Technology, #8242; Lot: 9)
phospho-ERK1/2 (Cell Signaling Technology, #9101S; Lot: 13)
ERK1/2 (Cell Signaling Technology, #9102; Lot: 7)
phospho-MEK1/2 (Cell Signaling Technology, #9121; Lot: 13)
MEK1/2 (Cell Signaling Technology, #9122; Lot: 14)
OASL (GeneTex, #GTX31572; Lot: 822201084)
rabbit polyclonal anti-mouse OASL1 (gift from our author Y.-J.K, PMID: 23416614)
eNOS (clone: D9A5L; Cell Signaling Technology, #32027; Lot: 3)
phospho-STAT1 (clone: D4A7; Cell Signaling Technology, #7649; Lot: 5)
STAT1 (clone: D1K9Y; Cell Signaling Technology, #14994; Lot: 5)
phospho-STAT2 (Cell Signaling Technology, #4441; Lot: 5)
STAT2 (clone: D9J7L; Cell Signaling Technology, #72604; Lot: 2)
phosphor-IRF7 (Invitrogen, #PA5-64834; Lot: XD3539523)
IRF7 (clone: SC0617; Invitrogen, #MA5-41165; Lot: XB3565156A)
phosphor-IRF3 (clone: 4D4G; Cell Signaling Technology, #4947; Lot: 13)
IRF3 (clone: D83B9; Cell Signaling Technology, #4302; Lot: 7)
Actin (Santa Cruz, #sc-1615)
GAPDH (GeneTex, #GTX100118; Lot: 43712)
Goat Anti-Rabbit IgG Antibody, Peroxidase Conjugated (EMD Millipore, #AP132P; Lot: 3766965)
Rabbit Anti-Goat IgG Antibody, HRP conjugate (EMD Millipore, #AP106P; Lot: 2803656)
Alexa Fluor™ 488 Goat anti-Rabbit IgG (H+L) (Invitrogen, #A-11008; Lot: 1829924)
Alexa Fluor™ 594 Goat anti-Rabbit IgG (H+L) (Invitrogen, #A-11012; Lot: 1844440)
Alexa Fluor™ 647 Goat anti-Rabbit IgG (H+L) (Invitrogen, #A-21245; Lot: 1863958)

Validation

All antibodies used in this study are commercially available and validated by the manufacturer of each antibody except rabbit polyclonal anti-mouse OASL1, which was developed and given by our author Y.-J.K. (PMID: 23416614): validated species: Mouse; application: WB, IP, IF
PerCP anti-mouse CD45 Antibody: validated species: Mouse; application: FC - Quality tested, https://www.biolegend.com/nl-be/products/percp-anti-mouse-cd45-antibody-4265
APC anti-mouse/human CD11b Antibody: validated for species: Mouse, Human, Cross-Reactivity: Chimpanzee, Baboon, Cynomolgus, Rhesus, Rabbit (Lapine); application: FC - Quality tested, https://www.biolegend.com/fr-fr/products/apc-anti-mouse-human-cd11b-antibody-345
Brilliant Violet 421™ anti-mouse CD64 (FcγRI) Antibody: validated species: Mouse; application: FC - Quality tested, https://www.biolegend.com/de-de/products/brilliant-violet-421-anti-mouse-cd64-fcgammari-antibody-8992
Brilliant Violet 650™ anti-mouse Ly-6G Antibody: validated species:Mouse; application: FC - Quality tested, https://www.biolegend.com/ja-jp/products/brilliant-violet-650-anti-mouse-ly-6g-antibody-11981
PE/Cyanine7 anti-mouse CD11c Antibody: validated species: Mouse; application: FC - Quality tested, https://www.biolegend.com/it-it/products/pe-cyanine7-anti-mouse-cd11c-antibody-3086
APC/Cyanine7 anti-mouse I-A/I-E Antibody: validated species: Mouse; application: FC - Quality tested, https://www.biolegend.com/

ja-jp/products/apc-cyanine7-anti-mouse-i-a-i-e-antibody-5966
Alexa Fluor® 488 anti-mouse CD31 Antibody: validated species: Mouse; application: Each lot of this antibody is quality control tested by immunofluorescent staining with flow cytometric analysis., https://www.biolegend.com/en-gb/products/alexa-fluor-488-anti-mouse-cd31-antibody-3093
Alexa Fluor® 594 anti-mouse CD31 Antibody: validated species: Mouse; application: FC, IHC-F - Quality tested, https://www.biolegend.com/en-gb/products/alexa-fluor-594-anti-mouse-cd31-antibody-9633
Anti-alpha smooth muscle Actin antibody: validated species: Mouse, Human; application: WB, IHC-P, https://www.abcam.com/alpha-smooth-muscle-actin-antibody-ab5694.html
Anti-CD31 antibody: validated species: Human; application: IHC-P, https://www.abcam.com/cd31-antibody-ab28364.html
Phospho-Akt (Ser473) (D9E) XP® Rabbit mAb: validated species: Human, Mouse, Rat, Hamster, Monkey, D. melanogaster, Zebrafish, Bovine; application: WB, IP, IHC, IF, Fc, https://www.cellsignal.com/products/primary-antibodies/phospho-akt-ser473-d9e-xp-rabbit-mab/4060
Akt Antibody: validated species: Human, Mouse, Rat, Hamster, Monkey, Chicken, D. melanogaster, Bovine, Dog, Pig, Guinea Pig; application: WB, IP, IF, Fc, https://www.cellsignal.com/products/primary-antibodies/akt-antibody/9272
Phospho-NF-κB p65 (Ser276) Antibody: validated species: Human, Mouse, Rat; application: WB, IHC, https://www.cellsignal.com/product/productDetail.jsp?productId=3037
NF-κB p65 (D14E12) XP® Rabbit mAb: validated species: Human, Mouse, Rat, Hamster, Monkey, Dog; application: WB, IP, IHC, IF, Fc, ChIP, C&R, https://www.cellsignal.com/products/primary-antibodies/nf-kb-p65-d14e12-xp-rabbit-mab/8242?site-search-type=Products&N=4294956287&Ntt=+8242%29&fromPage=plp&_requestid=4081307
Phospho-p44/42 MAPK (Erk1/2) (Thr202/Tyr204) Antibody: validated species: Human, Mouse, Rat, Hamster, Monkey, Mink, D. melanogaster, Zebrafish, Bovine, Pig, C. elegans; application: WB, IP, IF, Fc, https://www.cellsignal.com/products/primary-antibodies/phospho-p44-42-mapk-erk1-2-thr202-tyr204-antibody/9101
p44/42 MAPK (Erk1/2) Antibody: validated species: Human, Mouse, Rat, Hamster, Monkey, Mink, Zebrafish, Bovine, Pig, S. cerevisiae; application: WB, IP, IHC-P, https://www.cellsignal.com/products/primary-antibodies/p44-42-mapk-erk1-2-antibody/9102
Phospho-MEK1/2 (Ser217/221) Antibody: validated species: Human, Mouse, Rat, Monkey, S. cerevisiae; application: WB, IP, https://www.cellsignal.com/products/primary-antibodies/phospho-mek1-2-ser217-221-antibody/9121?site-search-type=Products&N=4294956287&Ntt=9121%29&fromPage=plp&_requestid=4081744
MEK1/2 Antibody: validated species: Human, Mouse, Rat, Monkey, D. melanogaster; application: WB, IP, https://www.cellsignal.com/products/primary-antibodies/mek1-2-antibody/9122
OASL antibody: validated species: Human, Mouse, Rat; application: WB, ICC/IF, ELISA, https://www.genetex.com/Product/Detail/OASL-antibody/GTX31572
eNOS (D9A5L) Rabbit mAb: validated species: Human, Mouse, Rat, Bovine; application: WB, IP, IF, https://www.cellsignal.com/products/primary-antibodies/enos-d9a5l-rabbit-mab/32027
Phospho-Stat1 (Tyr701) (D4A7) Rabbit mAb: validated species: Human, Mouse, Rat; application: WB, IP, IF, Fc, ChIP, https://www.cellsignal.com/products/primary-antibodies/phospho-stat1-tyr701-d4a7-rabbit-mab/7649
Stat1 (D1K9Y) Rabbit mAb: validated species: Human, Mouse, Rat, Monkey; application: WB, IP, IHC, IF, Fc, ChIP, https://www.cellsignal.com/products/primary-antibodies/stat1-d1k9y-rabbit-mab/14994
Phospho-Stat2 (Tyr690) Antibody: validated species: Human; application: WB, https://www.cellsignal.com/products/primary-antibodies/phospho-stat2-tyr690-antibody/4441?site-search-type=Products&N=4294956287&Ntt=4441%29&fromPage=plp&_requestid=4079863
Stat2 (D9J7L) Rabbit mAb: validated species: Human, Mouse; application: WB, IP, IF, Fc, ChIP, C&R, https://www.cellsignal.com/products/primary-antibodies/stat2-d9j7l-rabbit-mab/72604
Phospho-IRF7 (Ser477) Polyclonal Antibody: validated species: Human, Mouse, Rat; application: Western Blot (WB), https://www.thermofisher.com/antibody/product/Phospho-IRF7-Ser477-Antibody-Polyclonal/PA5-64834
IRF7 Recombinant Rabbit Monoclonal Antibody (SC0617): validated species: Human, Mouse, Rat, Zebrafish; application: WB, IHC-P, ICC/IF, Fc, https://www.thermofisher.com/antibody/product/IRF7-Antibody-clone-SC0617-Recombinant-Monoclonal/MA5-41165
Phospho-IRF-3 (Ser396) (4D4G) Rabbit mAb: validated species: Human, Mouse; application: WB, https://www.cellsignal.com/products/primary-antibodies/phospho-irf-3-ser396-4d4g-rabbit-mab/4947
IRF-3 (D83B9) Rabbit mAb: validated species: Human, Mouse, Rat, Monkey, application: WB, IP, https://www.cellsignal.com/products/primary-antibodies/irf-3-d83b9-rabbit-mab/4302
GAPDH antibody: validated species: Human, Mouse, Rat, Zebrafish, Yeast, Rabbit, Drosophila, Bovine, Dog, Hamster, Chicken, Pig, Monkey, Caenorhabditis elegans, E. coli, Mosquito, Nematode, Pika, Plant, Fish, Bacteria, milkfish, application: WB, ICC/IF, IHC-P, IP, https://www.genetex.com/Product/Detail/GAPDH-antibody/GTX100118
Actin (C-11): validated species: mouse, rat, human, Xenopus laevis, zebrafish and Caenorhabditis elegans; application: WB, IP, IF, Fc, ELISA, https://search.cosmobio.co.jp/cosmo_search_p/search_gate2/docs/SCB_/SC1615.20070227.pdf
Goat Anti-Rabbit IgG Antibody, Peroxidase Conjugated (EMD Millipore, AP132P): validated species: Rabbit; application: WB, IHC, ELISA, https://www.merckmillipore.com/KR/ko/product/Goat-Anti-Rabbit-IgG-Antibody-Peroxidase-Conjugated,MM_NF-AP132P
Rabbit Anti-Goat IgG Antibody, HRP conjugate (EMD Millipore, AP106P): validated species: Goat; application: ELISA, IHC, WB, https://www.merckmillipore.com/KR/ko/product/Rabbit-Anti-Goat-IgG-Antibody-HRP-conjugate,MM_NF-AP106P
Alexa Fluor™ 488 Goat anti-Rabbit IgG (H+L) (Invitrogen, A-11008): validated species:Rabbit; application: IHC, ICC/IF, Flow, https://www.thermofisher.com/antibody/product/Goat-anti-Rabbit-IgG-H-L-Cross-Adsorbed-Secondary-Antibody-Polyclonal/A-11008
Alexa Fluor™ 594 Goat anti-Rabbit IgG (H+L) (Invitrogen, A-11012): validated species: Rabbit; application: ICC/IF, Flow, https://www.thermofisher.com/antibody/product/Goat-anti-Rabbit-IgG-H-L-Cross-Adsorbed-Secondary-Antibody-Polyclonal/A-11012
Alexa Fluor™ 647 Goat anti-Rabbit IgG (H+L) (Invitrogen, A-21245): validated species: Rabbit; application: IHC, ICC/IF, https://www.thermofisher.com/antibody/product/A-21245.html

Eukaryotic cell lines

Policy information about cell lines

Cell line source(s)	Human umbilical vein endothelial cells (HUVECs; LONZA), Human aortic endothelial cells (HUAECs; Promocell), Human aortic smooth muscle cells (HASMCs; LONZA) were purchased. Mouse primary cells including MAEC and VSMC were isolated from

	aortas of 3 to 5-week-old mice. BMDM was isolated following the previously published protocol (PMID: 32883094).
Authentication	Cell lines used in this study were authenticated for marker expression by each manufacturer.
Mycoplasma contamination	All human cell lines were tested negative for mycoplasma contamination.
Commonly misidentified lines (See ICLAC register)	Commonly misidentified lines were not used in this study.

Animals and other organisms

Policy information about studies involving animals; ARRIVE guidelines recommended for reporting animal research

Laboratory animals	C57BL/6J (JAX stock #000664; B6/J), B6.129P2-Apoetm1Unc/J (JAX stock #002052; Apoe-/-), B6.Cg-Tg(Tek-cre)12Fiv/J (JAX stock #004128; Tie2Cre), B6.129P2-Lyz2tm1(cre)Ifo/J (JAX stock #004781; LysMCre) and B6.129P2-Nos3tm1Unc/J (JAX stock #002684; eNOS KO) strains were purchased from the Jackson Laboratory. Tg(Cdh5-cre/ERT2)#Ykub strain was kindly donated from Yoshiaki Kubota (KEIO UNIVERSITY). B6.129P2-Oasl1tm1Lms/J (Oasl1-/-) mice were previously generated (PMID: 23416614) and were backcrossed for more than 10 generations onto a C57BL/6J background. The B6.Oasl1tm1a(EUCOMM)Wtsi/BcmMmucd stain was purchased from the MMRRC. All mice used in vivo study were male and older than 8 weeks while female and male mice younger than 4 weeks of age were used for primary endothelial cell isolation, and female mice were used for bone-marrow isolation. All mice were provided water and food ad libitum and were housed under a 12-hour light/dark cycle (light, 07:00 to 19:00 hours) at 22°C ± 2°C with 55% ± 5% humidity
Wild animals	This study did not involve wild animals.
Field-collected samples	The study did not involve samples collected from the field.
Ethics oversight	All animal procedures were approved by the Institutional Animal Care and Use Committee (IACUC) of Ewha Womans University, Seoul, Korea (certification numbers: IACUC 19-004 and EWHA IACUC 21-065-1) and conformed to its regulations.

Note that full information on the approval of the study protocol must also be provided in the manuscript.

Human research participants

Policy information about studies involving human research participants

Population characteristics	Tissue sections of human thoracic and abdominal aortas from patients were used in this study. The state of samples: with or without plaques was determined by gross anatomy, then further validated by histological observation. Information of covariate-relevant characteristics including age over 50 years, diagnosis for atherosclerosis without other symptoms (hypertension, diabetes mellitus), and without statin treatment were concomitantly considered before being used for experiments.
Recruitment	All donors are patients who went to surgical operations, recruited and managed by Yonsei Severance Hospital. Written informed consent was obtained from all subjects.There was no selection bias.
Ethics oversight	Experiments using human samples in this study were reviewed and approved by the Institutional Review Board (IRB) of Yonsei Severance Hospital. Human samples were provided by Yonsei Severance Hospital (Seoul, Korea).

Note that full information on the approval of the study protocol must also be provided in the manuscript.

Flow Cytometry

Plots

Confirm that:

- [x] The axis labels state the marker and fluorochrome used (e.g. CD4-FITC).
- [x] The axis scales are clearly visible. Include numbers along axes only for bottom left plot of group (a 'group' is an analysis of identical markers).
- [x] All plots are contour plots with outliers or pseudocolor plots.
- [x] A numerical value for number of cells or percentage (with statistics) is provided.

Methodology

Sample preparation	After euthanizing the mice with CO_2, aortas were harvested and isolated into a single-cell suspension at 37 °C for 60 minutes by incubation with gentle shaking with Ca^{2+}/Mg^{2+} HBSS containing collagenase I (675 units/mL, Sigma-Aldrich, C0130), collagenase XI (187.5 units/mL, Sigma-Aldrich, C7657), hyaluronidase (90 units/mL, Sigma-Aldrich, H3884) and DNase I (90 units/mL, Sigma-Aldrich, DN25).
Instrument	FACSAria (for cell sorting, BD Biosciences), LSR Fortessa (for flow cytometry analysis, BD Biosciences)
Software	FlowJo (for data analysis, v 10.0.7, FlowJo, LLC)

Cell population abundance	For the lesional infiltrated cells, all of the cells isolated from atherosclerotic aorta were analyzed. After debris exclusion, all live aortic single-cells, without specific cell-type gating, were sorted. More than 70% of cells in total cells were live single-cells. Only dye for live/dead cell staining (DAPI) was used in cell sorting.
Gating strategy	FSC-A/SSC-A for debris removal, FSC-A/FSC-W for single-cell gating. The detailed gating strategy used in this study is described in Supplementary fig. 4. For single cell RNA sequencing, DAPI was used for live/dead cell staining and gating for live cell sorting.

☒ Tick this box to confirm that a figure exemplifying the gating strategy is provided in the Supplementary Information.
